# Postpandemic Evaluation of the Eco-Efficiency of Personal Protective Equipment Against COVID-19 in Emergency Departments: Proposal for a Mixed Methods Study

**DOI:** 10.2196/50682

**Published:** 2023-12-07

**Authors:** Simon Berthelot, Yves Longtin, Manuele Margni, Jason Robert Guertin, Annie LeBlanc, Tania Marx, Khadidiatou Mangou, Ariane Bluteau, Diego Mantovani, Sergey Mikhaylin, Frédéric Bergeron, Valérie Dancause, Anne Desjardins, Nadia Lahrichi, Danielle Martin, Charles Jérôme Sossa, Philippe Lachapelle, Isabelle Genest, Stéphane Schaal, Anne Gignac, Stéphane Tremblay, Éric Hufty, Lynda Bélanger, Erica Beatty

**Affiliations:** 1 Axe Santé des populations et pratiques optimales en santé Centre de recherche CHU de Québec-Université Laval Québec, QC Canada; 2 Département de médecine de famille et de médecine d'urgence Faculté de médecine Université Laval Québec, QC Canada; 3 Jewish General Hospital Montréal, QC Canada; 4 Ecole Polytechnique Université de Montréal Montréal, QC Canada; 5 Département de médecine sociale et préventive Faculté de médecine Université Laval Québec, QC Canada; 6 Services des urgences Centre hospitalier universitaire de Besançon Besançon France; 7 Axe Médecine régénératrice Centre de recherche CHU de Québec-Université Laval Québec, QC Canada; 8 EcoFoodLab Département des sciences de aliments, Institut sur la Nutrition et les Aliments Fonctionnels Université Laval Québec, QC Canada; 9 Bibliothèque Université Laval Québec, QC Canada; 10 CHU de Québec-Université Laval Québec, QC Canada; 11 Fashion Design and Creative Direction Toronto Metropolitan University Toronto, ON Canada; 12 Institut régional de santé publique Université d’Abomey-Calavi Ouidah Benin; 13 Département de médecine d’urgence Hôpital Montfort Ottawa, ON Canada

**Keywords:** COVID-19, SARS-CoV-2, personal protective equipment, emergency department, health care workers, systematic review, cost-consequence analysis, time-driven activity-based costing, life cycle assessment, ecological footprint

## Abstract

**Background:**

The COVID-19 pandemic has had a profound impact on emergency department (ED) care in Canada and around the world. To prevent transmission of COVID-19, personal protective equipment (PPE) was required for all ED care providers in contact with suspected cases. With mass vaccination and improvements in several infection prevention components, our hypothesis is that the risks of transmission of COVID-19 will be significantly reduced and that current PPE use will have economic and ecological consequences that exceed its anticipated benefits. Evidence is needed to evaluate PPE use so that recommendations can ensure the clinical, economic, and environmental efficiency (ie, eco-efficiency) of its use.

**Objective:**

To support the development of recommendations for the eco-efficient use of PPE, our research objectives are to (1) estimate the clinical effectiveness (reduced transmission, hospitalizations, mortality, and work absenteeism) of PPE against COVID-19 for health care workers; (2) estimate the financial cost of using PPE in the ED for the management of suspected or confirmed COVID-19 patients; and (3) estimate the ecological footprint of PPE use against COVID-19 in the ED.

**Methods:**

We will conduct a mixed method study to evaluate the eco-efficiency of PPE use in the 5 EDs of the CHU de Québec-Université Laval (Québec, Canada). To achieve our goals, the project will include four phases: systematic review of the literature to assess the clinical effectiveness of PPE (objective 1; phase 1); cost estimation of PPE use in the ED using a time-driven activity-based costing method (objective 2; phase 2); ecological footprint estimation of PPE use using a life cycle assessment approach (objective 3; phase 3); and cost-consequence analysis and focus groups (integration of objectives 1 to 3; phase 4).

**Results:**

The first 3 phases have started. The results of these phases will be available in 2023. Phase 4 will begin in 2023 and results will be available in 2024.

**Conclusions:**

While the benefits of PPE use are likely to diminish as health care workers’ immunity increases, it is important to assess its economic and ecological impacts to develop recommendations to guide its eco-efficient use.

**Trial Registration:**

PROSPERO CRD42022302598; https://www.crd.york.ac.uk/prospero/display_record.php?RecordID=302598

**International Registered Report Identifier (IRRID):**

DERR1-10.2196/50682

## Introduction

### Background

#### The Impact of the Pandemic on Emergency Departments

The COVID-19 pandemic has had a profound effect on emergency care in Canada and around the world. Emergency departments (EDs) have been reconfigured to meet the new demands of the pandemic [1]. Some of the reconfigurations include increased capacity, physical divisions into identified risk zones, changes in care protocols, redefinition of staff tasks, and development of protective measures against COVID-19 nosocomial transmission [2,3].

#### Personal Protective Equipment

In order to prevent transmission at the peak of the pandemic, personal protective equipment (PPE) was required for all ED care providers (eg, physicians, nurses, and nursing assistants) in contact with patients with proven or suspected COVID-19. PPE encompasses all clothing and other equipment used to protect health care workers from injury or infection [4], and is considered the last step in the hierarchy of controls in infection prevention [5,6]. With respect to the COVID-19 pandemic, the term PPE refers primarily to two options (see Multimedia Appendices 1 and 2) [7]: (1) standard PPE, which includes procedural masks, isolation gowns, nonsterile gloves, and ocular protection; and (2) enhanced PPE which includes respirators (eg, N95), water-repellent gowns, long-cuff nitrile procedure gloves, and face shields. Since the beginning of the pandemic, the understanding of the COVID-19 modes of transmission and the most effective equipment to protect against it have greatly evolved. In many jurisdictions, enhanced PPE was initially reserved for aerosol-generating procedures (eg, endotracheal intubation), that is, aerosolized particles of <5 μm that can remain suspended in the air for extended periods of time and circulate over long distances on air currents [8]. In Canada, in 2021, the use of enhanced PPE with a respirator has gradually become the norm when caring for patients with confirmed or suspected COVID-19 cases, even in the absence of aerosol-generating procedures [9-11].

#### Conflicting Rules for PPE Use

The rules for PPE use in health care settings have fluctuated greatly and have been a source of uncertainty [8,12]. Often developed as a compromise between incomplete scientific evidence [7,8,13], uncertain PPE supplies [14-16], and staff concerns [12,17], these rules were sometimes so confusing for health care workers that their trust towards the infection control teams and managers was diminished [17]. Although the benefits of PPE for protecting health care workers against SARS-CoV-2 have been demonstrated [7,13,18], the level of evidence is relatively low and its use is more or less influenced by subjective preferences and personal values [19].

#### New Context, New Rules?

Although the achievement of a certain level of immunity in the population provides significant protection against severe or chronic symptoms of COVID-19 [20], important questions remain; what types of PPE will be needed to protect health care workers as SARS-CoV-2 becomes endemic and circulation in the community continues unabated? Somewhat less discussed since the beginning of the pandemic, the societal consequences of PPE use must also be considered. What will be the opportunity cost for public institutions to pursue the expanded use of PPE [21-23]? What is the ecological cost of continuing on this path [24-27]? More than ever, evidence is needed to evaluate PPE use and to provide recommendations that ensure the clinical, economical, and ecological efficiency of its use.

### Hypothesis and Research Goal

With mass vaccination and improvements in several infection prevention components included in the hierarchy of controls, our hypothesis is that the risk of transmission of COVID-19 will be significantly reduced and that wearing fully enhanced PPE for every suspected COVID-19 patient will have economic and ecological consequences that exceed the anticipated benefits. To support the development of optimal rules for PPE use, our research objectives for this project are to (1) determine the clinical effectiveness (reduced transmission, hospitalization, mortality, and work absenteeism) of PPE to protect immune and nonimmune health care workers against COVID-19; (2) estimate the financial cost of using PPE in the ED for the management of patients with suspected or confirmed COVID-19; and (3) estimate the ecological footprint of PPE use against COVID-19 in the ED.

### Theoretical Framework

Our objectives address a transdisciplinary subject that goes beyond the boundaries of health research and consequently requires a conceptual framework that aggregates complementary theoretical approaches. We therefore propose an extended application of the value-based health care framework, which consists of orienting care practices, decisions, and policies to obtain the best health outcomes at the lowest cost. This model, developed by Porter [28-30] in the 2000s, is now widely used by various organizations [31-35] to improve health services. Optimizing the value of care can, among other things, be achieved by reducing its costs, which can be minimized by eliminating the inappropriate use of resources. The notion of value thus overlaps with the notion of eco-efficiency [36]. The Organization for Economic Cooperation and Development defines it as “a management philosophy that encourages business to seek environmental improvements that are accompanied by economic benefits” [37,38]. Eco-efficiency is closely tied to the appropriate use of resources extracted from the environment and the emissions generated throughout the life cycle in order to increase the economic value of what is ultimately produced. By revisiting these 2 concepts for our research framework, we will define eco-efficiency in health care as the maximization of the clinical benefits of a health care activity while minimizing its costs and its ecological footprint.

## Methods

### Setting

The project will evaluate the eco-efficiency of PPE use in the 5 EDs of the CHU de Québec-Université Laval (hereafter the CHU) in Québec City (Canada). The CHU is the largest academic hospital in Québec and 1 of the 3 largest in Canada. Its EDs record nearly 240,000 visits annually. They form the same clinical department, use the same protocols, and offer similar care paths. In terms of PPE, the donning and doffing protocols are identical. All CHU wards use disposable PPE. At the beginning of the pandemic, all CHU EDs cleaned and reused protection goggles and face shields. Similarly, one ED site used reusable gowns but has since switched to fully disposable PPE (Multimedia Appendix 3). The CHU’s EDs are an ideal setting to conduct the proposed study because (1) several thousand units of PPE are used daily; (2) each of the 5 EDs has cold (non–COVID-19), warm (suspected COVID-19 cases), and hot (confirmed COVID-19 cases) areas; (3) there is a large diversity of patients, from children to elderly, and both medical and surgical cases; and (4) as opposed to high-risk units such as intensive care and COVID-19 units, where infected patients are grouped together and where the wearing of PPE appears more indicated, the majority of patients evaluated in the ED do not have COVID-19, even when the clinical picture is compatible. It is therefore in the ED that an evaluation of the eco-efficiency of PPE use can have the greatest impact on the recommendations for use in a low-risk clientele. Furthermore, since donning and doffing protocols do not differ significantly from one department to another and are very similar in all institutions in Quebec and across Canada, we will be able to infer some of our results from the entire hospital reality in Canada.

### Study Design

We propose a mixed methods, multiphase study, merging approaches from epidemiology, biostatistics, industrial engineering, accounting, health economics, mathematics, psychology, and environmental engineering. To achieve our goals, the project will include four phases: systematic review of the literature to assess the clinical benefits of PPE (objective 1; phase 1); cost estimation of PPE use in the ED using a time-driven activity-based costing method (objective 2; phase 2); ecological footprint estimation of wearing PPE during the pandemic using a life cycle assessment approach (objective 3; phase 3); and cost-consequence analysis and focus groups (integration of objectives 1 to 3; phase 4).

For each of these phases, we will propose analyses distinguishing standard and enhanced PPE.

### Phase 1: Systematic Review of the Literature (Objective 1)

#### Protocol and Registration

The protocol for this review is registered in the PROSPERO database of systematic reviews (CRD42022302598) and we will follow recommendations of the PRISMA (Preferred Reporting Items for Systematic Reviews and Meta-Analysis) guidelines for its reporting [39].

#### Research Question

What effect does wearing PPE have on health care workers’ risk of becoming infected with SARS-CoV-2, being secondarily absent from work, admitted to the hospital or to the intensive care unit (ICU), or die? Table 1 shows the PICOS framework for the systematic review.

**Table 1:** PICOS (population, intervention, comparison, outcomes, and study design) framework for the systematic review.

Population	Health care workers
Intervention	Standard or enhanced personal protective equipment
Comparison	No or incomplete personal protective equipment
Outcomes	SARS-CoV-2 (COVID-19) infection, hospital admission, intensive care unit admission, mortality, and work absenteeism
Study design	Experimental or observational studies

#### Search Strategy

We will search for studies published since December 2019 (identification of SARS-CoV-2 in Wuhan) comparing health care workers’ use of disposable or reusable, standard or enhanced PPE to no protection used or a different combination of PPE components. We will use Medline (Ovid), Embase, Cochrane Library, CINAHL, Epistemonikos, ClinicalTrials.gov, MedRxiv, and Web of Science search engines, without language restrictions. The search strategy was developed with the assistance of a qualified librarian and is available in the appendices (Multimedia Appendix 3). This strategy will be executed three times: (1) at the start of the systematic review (May 2022); (2) at the end of the prepublication data extraction (June 2023); and (3) before phase 4 (January 2024) to update the meta-analysis to adequately inform our focus groups.

#### Article Selection and Eligibility Criteria

Using the Covidence systematic review software (Veritas Health Innovation), 2 independent reviewers will select abstracts, read the articles, and determine their eligibility. All experimental or observational studies (cohort, case-control, or cross-sectional studies) comparing the use of PPE with the absence of PPE with regard to health care workers’ risk of SARS-CoV-2 infection, hospitalization (ward or ICU), death, or absence from work, will be included in the review. Studies evaluating the effect of PPE when worn by nonhealth workers, narrative reviews, editorials, modeling studies without original clinical data, and practice guidelines will be excluded. Systematic reviews will also be excluded but will be consulted to identify original articles potentially missed by our search strategy. Bibliographies of selected articles will be reviewed in the same manner.

#### Data Extraction and Quality Assessment of Selected Studies

In total, 2 independent reviewers will collect data from the selected articles using a previously tested extraction grid. From each article, they will extract, when available, the following variables: authors; title; date and scientific journal of publication; country where the research was conducted; study design; population studied; sample size; age; gender; sex; vaccination or immune status; and comorbidities of the participants, study outcomes, and results. The quality of the selected studies will be assessed by 2 reviewers using the Cochrane Risk of Bias Tool for randomized studies [40] and ROBINS-I for observational studies [41].

#### Disagreements Among Reviewers

Disagreement between reviewers will be resolved through discussion and consensus. If a disagreement persists, a third reviewer will mediate to reach a final consensus.

#### Analysis and Synthesis of Results

The results will be presented in tabular and narrative form and will compare the benefits of standard or enhanced PPE to the absence of PPE use. Based on previous systematic reviews, we know that a random effects model meta-analysis is feasible and will then be conducted. According to the original data available, we will calculate estimates of relative risks (RR) or odds ratios (OR) on the risk of transmission of SARS-CoV-2 and of the other outcomes evaluated. The necessary numbers of PPE that must be used (number needed to treat) to prevent one SARS-CoV-2 transmission, admission, or death among staff will be calculated secondarily. Randomized studies will be analyzed separately from observational studies, as recommended by the Cochrane Handbook [42,43]. If studies are in sufficient numbers, we will conduct subgroup analyses by analyzing separately (1) the studies conducted in EDs; and (2) the studies conducted after the start of vaccination in the country where the research took place. Heterogeneity between studies will be assessed using the *I*^2^, a statistic that estimates the percentage of variation in results between studies that are not explained by chance. Thus, an *I*^2^ value between 0 and 40% will be considered to represent a low level of heterogeneity, while values between 30% and 60% represent a moderate level, between 50% and 90% a substantial level, and between 75% and 100% a considerable level [44]. The causes of heterogeneity will be examined by stratifying our outcome measures by (1) the country where the studies were conducted and (2) the clinical departments or units from which the data originated (eg, COVID-19 unit and ICU). We will also conduct a sensitivity analysis excluding studies with a high risk of bias. Finally, the quality of evidence for each outcome (high, moderate, low, and very low) will be assessed using the GRADE (Grading of Recommendations Assessment, Development, and Evaluation) approach [45].

### Phase 2: Cost Estimation of PPE Use (Objective 2)

#### Concept

The cost of PPE use goes beyond the cost of purchasing its components. Wearing PPE means donning and doffing by care providers who, during this time, cannot engage in direct care, which is a cost to the health care system. Using PPE also incurs costs for material disposal (eg, garbage collection) and overhead (eg, supply management). Therefore, we will estimate the mean cost of wearing PPE in the CHU for contact with a possible case of COVID-19 using a time-driven activity-based costing method [46,47]. This method accounts for all expenditures incurred during direct patient care (eg, staff salaries and medical costs), consumables (eg, masks), and management costs (eg, disinfection service). It uses the duration of care processes to estimate the associated cost; the longer a process (eg, donning PPE) takes to be performed, the higher the cost. It provides a simpler, more accurate, and more reliable way of estimating the cost of health services than other methods frequently used in research or management, such as diagnosis-related group methods (DRG), the level of relative intensity of resource use (NIRRU) or the conventional activity-based costing method (ABC) [48-50]. Time-driven activity-based costing was previously used in many health care settings [48,51-56] and our team successfully adapted it for use in the ED [57-59]. For this project, we will apply this costing method to the care pathway of patients suspected with COVID-19 in the ED. We will analyze the processes involved in PPE use and disposal. The disposal component will include both trajectories of completely disposable PPE versus reusable gowns, as one of our sites used reusable gowns at the beginning of the pandemic. Cleaning of the reusable gowns was done by a subcontractor (Partagec), as such, the cleaning cost will be estimated based on the weight of the gowns used as stipulated in the contract with Partagec.

#### Method Steps

##### Overview

Time-driven activity-based costing essentially requires two parameters: (1) the cost per minute for each human or material resource involved in care, and (2) the duration in minutes of each care process. To derive these, we will use data from the fiscal year beginning April 1, 2020, through March 31, 2021. We will estimate the average costs of standard and enhanced PPE using the following steps.

##### Process Maps

We will first map the ED care pathway of suspected COVID-19 cases. We will also map all specific processes performed in the wearing, disposal, recycling, and disinfection of PPE. This will primarily include (1) donning, (2) doffing, (3) disposal, and (4) collection and disinfection or cleaning of reusable components.

##### Time Measurements

We will estimate the mean time required to complete each mapped process through prospective field measurements at the ED using a time-motion study software (UmtPlus Max, Laubrass).

##### Resource Costs per Minute

We will calculate the cost per minute (CAD $ per minute; with an average currency exchange rate of CAD $1=US $0.8) of each human or material resource identified in the process maps by dividing its total annual cost for the year 2020-21 by the number of minutes in the same year that the resource was available for care or service. The following is an example from our previous work.



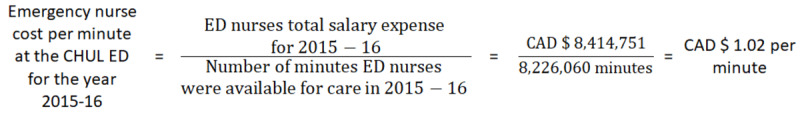



If applicable, a similar calculation will be performed for equipment by considering depreciation and maintenance expenses in the numerator and the number of minutes in service in 2020-21 in the denominator. The cost per minute of emergency physicians will be estimated by the same formula, but the total annual medical expenditures will be estimated using the average annual earnings of an emergency physician as obtained from the regulatory medical associations.

##### Consumable Costs

We will calculate from the CHU accounting records the average unit cost (CAD $/unit) of each component (gown, gloves, masks, and ocular protection) of PPE purchased by CHU in the financial year 2020-2021 and then break down the specific costs by supplier. The reusable equipment purchase cost will be amortized over the anticipated number of uses.

##### Overhead Expenses per Minute

Following a formula similar to item 3, we will calculate the cost per minute (CAD $ per minute) of overhead incurred by the CHU for PPE use (eg, rental or transportation of waste containers), by dividing the total overhead related to PPE use in the ED for the year 2020-21 by the number of minutes available for the care of the staff using PPE as part of their work.

##### Mean Process Costs

We will estimate the mean process costs for PPE by summing the costs of resources, consumables, and overhead according to the calculation presented in the following example based on previous work data and preliminary estimates. Similar calculations will be performed for all PPE-related processes (eg, doffing).



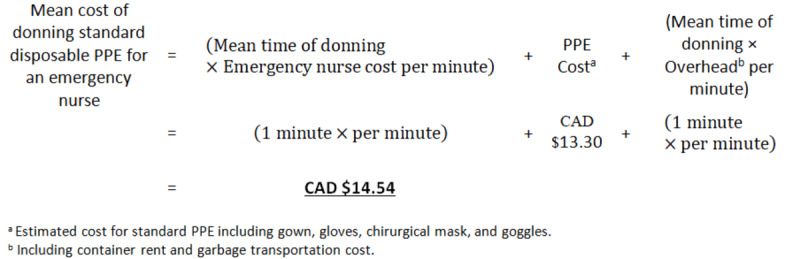



Further details on the method and its practical application can be found elsewhere [60,61].

#### Analyses

The preceding steps will estimate for each category of ED care providers (eg, nurses and physicians) the mean cost incurred during the pandemic for one standard and one enhanced PPE use, which includes donning, doffing, disposing, and disinfection or cleaning. A set of subanalyses will then estimate (1) the minimum and maximum costs of PPE use based on the minimum and maximum supplier unit costs of the PPE components purchased by the CHU in 2020-2021; (2) the weighted mean cost of PPE use based on the weight of participation of each category of providers in the care pathway, estimated using the mapping of a typical care pathway for COVID-19 in the ED (Multimedia Appendix 4); (3) The total cost of PPE use in the CHU EDs based on the number of suspected COVID-19 cases that consulted since the start of the pandemic, the weighted mean cost of using PPE, and the number of contacts with PPE per episode of care estimated with the mapping; and (4) mean costs based on component costs in the year prior to the pandemic (financial year 2019-2020) to estimate the effect of the shortage on equipment selling prices.

### Phase 3: Ecological Footprint Estimation of PPE Use (Objective 3)

#### Concept

The ecological footprint of standard and enhanced PPE will be estimated through a life cycle assessment that will measure the pollutant load of all material and energy resources used (inputs) and residual materials generated (outputs) by their use (Multimedia Appendix 5 for a typical life cycle diagram). The analysis will assess the environmental impact of the consumables (eg, gloves) and processes (eg, disinfection of face shields) required for PPE use in CHU EDs from cradle to grave, that is, from their design and manufacturing to their final disposal. The life cycle assessment methodology, which is well established in the industrial field, has previously been used in the health field as well [62-64].

#### Methodology Phases

##### Overview

The life cycle assessment will follow the ISO-14040/ISO-14044 standards governed by the International Organization for Standardization [65-67]. These international standards are developed through consensus among experts from industry, consumer associations, governments, nongovernmental organizations, and universities. The standards that will be used for this project provide a framework for an environmental management system, specifically the realization of a life cycle assessment. We will take the following steps.

##### Definition of the Objectives and Scope of the Study

The analysis will aim to assess the ecological footprint of one PPE used by a care provider in the ED for contact with a potential or confirmed COVID-19 case (functional unit). We will compare the ecological footprints of standard and enhanced PPE assuming an uneven level of protection that will be measured with the systematic review from the previous phase. Since supplies fluctuated over the course of the pandemic resulting in a very wide variety of equipment at the CHU, we will limit our analyses to those PPE components that were (1) the most frequently used, (2) the most and least costly, and (3) those whose supply contracts predict their predominant use for the foreseeable future.

##### Emissions and Extractions Inventory

Using the previously developed mappings, we will break down the donning and doffing process as well as the upstream (eg, production) and downstream (eg, disposal) processes to identify the use of consumable or reusable equipment and materials, and their disposition to waste or disinfection units. An inventory of activities and flow of materials, energy, and services will be conducted using data obtained from the CHU and PPE suppliers (eg, raw materials for PPE components and energy consumed at the production plant) and will be scaled to the functional unit level, that is, the use of a single PPE. This inventory will quantify the pollutants emitted and the resources extracted over the entire life cycle using a life cycle database (ecoinvent database, version 3.9.1; ecoinvent) adapted to Québec and data available in the life-cycle assessment literature.

##### Environmental Impact Analysis

Using the open access software OpenLCA, (version 1.11.0; GreenDelta) each previously collected inventory data on extractions and emissions from PPE use will be translated into potential impacts on environmental issues such as (1) climate change (in kg of CO_2_ equivalent emitted), (2) human health (in disability-adjusted life years [DALYs]) [68], (3) resource use (in megajoules of energy—MJ), and (4) ecosystem quality (in PDF∙m^2^∙year, the fraction of species potentially lost from a given area in a year). Conversion of PPE inventory data into impact units for each damage category will be done by the validated IMPACTWorld+ assessment method [67,69]. This method allows for a regionalized analysis by providing continent-specific impact data. An impact measure reflects a sequence of intermediate effects of a product (eg, PPE) production, use, and disposal that, by polluting water, air, soil, or food for example, ultimately affect the environment or human health.

##### Results Interpretation

The impact results will be interpreted in relation to the functional unit, that is, the use of a single PPE for contact with a suspected COVID-19 case by a care provider. The critical points, that is, the elements of PPE use that account for the greatest proportion of its impact, will be identified. Sensitivity analyses will be conducted to assess the possibility of bias due to incomplete or inaccurate inventory data. Where calculations are performed using proxies from inventory databases rather than original supplier data, we will reanalyze these uncertain variables by applying values for worst- and best-case scenarios.

### Phase 4: Cost-Consequence Analysis and Focus Groups (Integration of Objectives 1 to 3)

#### Cost-Consequence Analysis

The results of the analyses of the previous 3 phases on benefits (OR, RR, and number needed to treat), costs, and ecological footprint will be scaled to a single PPE use for contact with a suspected or confirmed COVID-19 case by a care provider in the ED. They will also be secondarily extrapolated for the full duration of the pandemic from the number of suspected COVID-19 cases assessed in the CHU EDs during this period. Using data from the Institut national de santé publique du Québec on the hospitalization rate of COVID-19 cases in Quebec [70] and the cost of an average hospitalization at the CHU obtained by the Cost per Care Pathway and Service (CPSS) system [71], we will estimate the potentially avoided costs by preventing hospitalizations through PPE. We will do the same by estimating the avoided costs related to staff absenteeism using a human capital approach [72] on the basis of the care providers’ salaries identified in phase 2. A cost-consequence analysis will then be carried out by presenting, in parallel, the benefits, costs, and environmental consequences with their 95% CIs in a simple and meaningful disaggregated format. A cost-consequence analysis is a type of economic evaluation that assesses a wide range of costs and consequences associated with an intervention. It can include all types of positive or negative effects of that intervention, including those that are health-related (eg, risk of transmission) and those that are not directly health-related (eg, environmental impact). By presenting the results separately in a nonaggregated manner, it allows stakeholders to determine for themselves the weight and value assigned to the costs and consequences presented for informed decision-making. We believe that cost-consequence analysis is most appropriate for our project because (1) the values and preferences of the stakeholders involved in PPE use are multiple and often divergent, making aggregative ratios or calculations inappropriate (eg the incremental cost-effectiveness ratio [ICER] assesses only one outcome or one benefit) and (2) the economic value of the benefits and consequences of PPE is not clearly elucidated, making cost-benefit analysis (ie, an analysis where all the consequences of intervention are converted into monetary value) problematic.

#### Focus Groups

The cost-consequence analysis will be presented and discussed in 4 focus groups [73] of 8 participants each [74], via 2 one-hour videoconferences for each group led by a facilitator and a note taker. A third videoconference will be held if not all topics have been covered in the first two. Individual interviews will be offered to participants who prefer this approach or who are unable to attend group meetings. The first group will bring together health care managers responsible for budgets; the second will bring together experts responsible for developing recommendations on PPE use in hospitals; the third will gather the perceptions of health care providers, nursing assistants, respiratory therapists, and physicians; and the last group will survey the perspective of patients and citizens. Additional groups of participants will be recruited as needed until the data are saturated. A purposeful sampling strategy will be used to ensure representation of various levels of authority (departmental, regional, local), professions (eg, nurses), regions (urban, semiurban, and rural), and individual characteristics (gender, ethnicity, and age) [75]. Participants will be presented with all the results in a simple, adapted format, after consultation with experts in content (eg, economists and environmental engineers) and information formatting (eg, information designers and knowledge transfer experts). The discussions in each group will be semistructured. The primary objectives of the semistructured discussions will be to (1) analyze the perspective of each group on the study results and (2) identify the cost-consequence analysis results (benefits vs costs vs environmental impact) that participants believe should be prioritized for future recommendations regarding PPE use in the ED. The meetings will be recorded and fully transcribed (verbatim). In total, 2 independent reviewers will code the responses on NVivo 12 (Lumivero) to structure the data and bring out the main themes. Their disagreements will be reconciled by discussion or, if necessary, by the intervention of a third researcher. The analysis will take an inductive approach to capture the different perspectives on PPE use in the ED from the data collected and then develop a theoretical framework.

### Ethical Considerations

This trial was approved by the CHU de Québec-Université Laval Research Ethics Board (#2022-6048).

### Dissemination Plan

Our dissemination plan has an integrated approach by including co-researchers and knowledge users in strategic positions in our team, including a member of the Québec Nosocomial Infection Committee responsible for defining the rules for the use of PPE (YL) and at the CHU, the infection prevention and control program managers (AD and VD), the assistant director (AG) and the consultant (SC) responsible for sustainable development, the nursing (ST) and medical (IG) managers of the EDs, the clinical and organizational performance director (PL), and the patient experience and partnership expertise office manager (LB). In total, 3 clinicians (IG, EEB, and a collaborator Éric Notebaert) and 2 patient partners (EH and a collaborator Jean-Pierre Gendreau) also participated in our process. All these people have contributed to the development of the concept, will participate in its implementation, will be informed of the results, will interpret them from their perspectives, and will be able to quickly integrate them into their practices. Our dissemination strategy will also include (1) articles in general information newspapers to publicize our initiative; (2) a website to present our team, our results, and our future projects; (3) presentations for the general public in citizen forums or congresses; (4) at least 6 presentations in national (Association des médecins d’urgence du Québec, Association des médecins microbiologistes infectiologues du Québec, Canadian Association of Emergency Physicians, and Canadian Association for Health Services and Policy Research) and international (Society for Academic Emergency Medicine and Infectious Diseases Society of America) scientific conferences; (5) at least 4 publications in recognized peer-reviewed scientific journals; and (6) presentations to our partner organizations, including primarily the Québec Nosocomial Infection Committee, which we plan to meet at least 4 times during the course of the project to share our preliminary results and progress.

## Results

### Study Preparation

We have assembled a very strong research team composed of patients, clinicians, administrators, and researchers. In addition, 2 patient partners met with us regularly and provided helpful comments to make our research plan patient-centered. From previous studies, our team was able to perform life cycle assessment [76-80] and cost-effectiveness [60,61,81,82] measures. This previous work will help with the proposed study.

### Phase 1 

Our search strategy for the systematic review, launched on May 10, 2022, and updated on June 7, 2023, has generated a list of 26,591 articles, of which 10,209 duplicates were removed. As of July 9, 2023, a total of 15,539 studies have been screened. Of these, 192 abstracts were retained for full-text review and 74 studies were included.

### Phase 2

Our team has made a first draft of the process map of the COVID-19 care pathway in the ED (Multimedia Appendix 6).

Process time measurements were performed in one of the CHU EDs for the donning and doffing of a PPE. Table 2 shows the mean time estimates for the different processes of PPE use. Our team is currently completing financial data collection on care providers’ salaries, labor hours, PPE component costs, and overhead. Of these data, the mean total cost of the components for a single use of standard and enhanced PPE was estimated to be CAD $13.30 and CAD $21.50 (US $9.79 and US $15.83), respectively.

**Table 2:** Mean donning and doffing time (minutes) for PPE^a^ based on field measurements.

Protective equipment	Donning			Doffing	
	n (%)	Mean (SD)	n (%)		Mean (SD)
Standard PPE^a^	63 (60)	1.28 (0.71)	65 (79)		1.10 (0.85)
Enhanced PPE	42 (40)	1.18 (0.43)	17 (21)		0.35 (0.37)
Any PPE	105 (100)	1.19 (0.45)	82 (100)		0.95 (0.84)

^a^PPE: personal protective equipment.

### Phase 3 

To date, we have begun an inventory of the materials used for each component using attenuated total reflectance-infrared spectroscopy. Additional analyses are performed to further validate the composition of the PPE components in terms of raw materials and the weight of each item. Once these data are obtained, the conversion of inventory into impact categories will be performed and analyzed.

### Protocol Endorsement

Our protocol has been endorsed by organizations dedicated to achieving sustainable and eco-efficient health care (Association québécoises des médecins pour l’environnement, CHU de Québec-Université Laval, INSPQ, Ministère de la santé et des services sociaux du Québec, PULSAR). We also have support for our research initiative from an organization in sustainable development (Nature Québec), with life cycle assessment expertise (the International Reference Center for Life Cycle of Products, Processes and Services—CIRAIG) and with national responsibility for the use of PPE (Québec Nosocomial Infection Committee). This broad support demonstrates the importance of addressing the eco-efficiency of health care, particularly the issue of PPE use.

### Research Agenda

We propose a 3-year research plan. After receiving funding on October 1, 2021, we have begun the administrative steps, mainly obtaining authorization from the ethics committee and intrainstitutional agreements for access to medico-administrative data. Phase 1 (systematic review) will run from May 2022 to September 2023 (16,401 abstracts screened) and will end with the submission of a paper for publication in December 2023. Phase 2 (cost estimation) and 3 (life cycle assessment) have begun in June 2022, and are expected to be completed in December 2023. Those 2 phases are performed concomitantly as they overlap in process mapping and medical-administrative data collection. Phase 4 will be conducted from January 2024 (preparation) to August 2024 end of focus group phase). Final publications and knowledge transfer activities will be completed in the last 6 months of 2024.

## Discussion

### Overview

PPE is the last step in the hierarchy of controls in infection prevention, but it has become an essential component of direct care for ED patients potentially infected with COVID-19. As new infection control measures have emerged, primarily from mass vaccination, some advocate for a more eco-efficient use of PPE, that is, one that provides the best possible protection with the least economic and ecological impact. The evidence generated by this study will support the infection control and prevention authorities in Canada and abroad by providing informed guidance for eco-efficient use of PPE in low-risk environments. They will also help us understand the perspective of different stakeholders on this sensitive and fundamental issue where material shortages, the risks to health care staff, the sustainability of the health system, and the protection of the environment are all intertwined.

Beyond this project on PPE, our multidisciplinary team of researchers from different backgrounds aims to initiate a profound change in managerial culture focused on sustainable health within our institutions, particularly in the ED. We propose an innovative conceptual framework, eco-efficiency in health, through which we wish to contribute to the best possible health outcomes in emergency care at the lowest cost and with the least environmental impact. Canada’s health care system is among the least eco-efficient in the world, and its environmental impact paradoxically affects Canadians’ health [83,84]. A paradigm shift is needed. Unfortunately, clinicians and managers have few tools to understand and act on their services’ ecological footprint. Our work incorporating life cycle assessment will help them to (1) provide the best care with the least environmental impact, (2) set realistic targets for reducing their ecological footprint, and (3) optimize interventions to achieve this. Our proposed project on PPE is in fact only the first in a series of analyses of different emergency care activities with the ultimate goal of proposing a model for eco-efficient emergency services [85].

### Challenges and Mitigation Strategies

Our project poses a few notable challenges. First, the systematic review will aim to estimate the benefits of using PPE as the risk of SARS-CoV-2 transmission decreases significantly as vaccination is accelerated. The relevance of the results of this phase will depend on the publication of articles that have evaluated the efficacy of PPE in a setting where the population and health care workers have been vaccinated. We have scheduled an update of our systematic review for September 2023, just before the start of our Phase 4 focus groups. Considering the wealth of scientific output on COVID-19 over the past years, we believe it unlikely that by this update, there will be no studies that have assessed the benefits of PPE since the beginning of vaccination. If not, we will use the results of our systematic review for nonimmune personnel and extrapolate the number needed to treat based on the observed rates of vaccine protection in the population. Second, our study will be conducted in only one institution, the CHU. However, since supply purchases in Québec are carried out from the same supplier for a group of establishments (eg, eastern Québec), both the costs and the ecological footprint of PPE use in the ED should reflect the reality of all Québec establishments. Similarly, since most PPE suppliers export their products around the world, the results of our analyses will be useful for other health authorities outside Québec. Third, with respect to the life cycle assessment, suppliers of the PPE components studied may not disclose certain information related to the composition of their products and their factory production. In the absence of such data, we will make estimates and use equivalencies from the literature and available life cycle assessment inventory databases. We will disclose the details of these approximations and the uncertainty of the results to transparently inform the resulting organizational decisions.

### Conclusions

The World Health Organization recently declared the end of the COVID-19 global health emergency, but acute and emergency care may maintain some habits and behaviors developed and implemented during this health crisis. The intensive use of PPE by care providers to protect patients and themselves from COVID-19 may be one of the pandemic care activities that will require further consideration. While the benefits of PPE use are likely to diminish as health care workers’ immunity increases, it is important to assess its economic and ecological consequences so that new parameters and recommendations can be developed to guide its use and ensure eco-efficiency now and in the future.

## Appendix

Multimedia Appendix 1 Standard personal protective equipment.

Multimedia Appendix 2 Enhanced personal protective equipment.

Multimedia Appendix 3 Literature search strategy.

Multimedia Appendix 4 Mapping of care processes and use of personal protective equipment in the emergency department for patients with COVID-19 (influenza-like illness).

Multimedia Appendix 5 Life cycle figure.

Multimedia Appendix 6 Canadian Institutes of Health Research grant evaluation.

## Abbreviations

ABC: activity-based costing
CIRAIG: International Reference Center for Life Cycle of Products, Processes and Services
CPSS: Cost per Care Pathway and Service
DALY: disability-adjusted life year
DRG: diagnosis-related group
ED: emergency department
GRADE: Grading of Recommendations Assessment, Development, and Evaluation
ICER: incremental cost-effectiveness ratio
ICU: intensive care unit
OR: odds ratios
PICOS: population, intervention, comparison, outcomes, and study design
PPE: personal protective equipment
PRISMA: Preferred Reporting Items for Systematic Reviews and Meta-Analysis
RR: relative risks

## Data Availability

The data sets generated or analyzed during this study are available from the corresponding author upon reasonable request.

## References

[1] Boyle AA, Henderson K (2020). COVID-19: resetting ED care. Emerg Med J.

[2] (2020). Global response to COVID-19 for emergency healthcare systems and providers: from the IFEM task force on ED crowding and access block. International Federation of Emergency Medicine.

[3] Frost DW, Shah R, Melvin L, de Juana MG, MacMillan TE, Abdelhalim T, Lai A, Rawal S, Cavalcanti RB (2020). Principles for clinical care of patients with COVID-19 on medical units. CMAJ.

[4] Patel A, D'Alessandro MM, Ireland KJ, Burel WG, Wencil EB, Rasmussen SA (2017). Personal protective equipment supply chain: lessons learned from recent public health emergency responses. Health Secur.

[5] (2015). Hierarchy of controls. Centers fo Disease Control and Prevention.

[6] (2020). Hierarchie des mesures de controle en milieu de travail. Institut National de Santé Publique du Québec.

[7] Verbeek JH, Rajamaki B, Ijaz S, Sauni R, Toomey E, Blackwood B, Tikka C, Ruotsalainen JH, Balci FSK (2020). Personal protective equipment for preventing highly infectious diseases due to exposure to contaminated body fluids in healthcare staff. Cochrane Database Syst Rev.

[8] Zhang XS, Duchaine C (2020). SARS-CoV-2 and health care worker protection in low-risk settings: a review of modes of transmission and a novel airborne model involving inhalable particles. Clin Microbiol Rev.

[9] (2021). La CNESST oblige également le port du N95, ou d'une protection supérieure, en zone tiède. CNESST.

[10] (2021). COVID-19 – La CNESST oblige le port du N95 ou d’une protection supérieure en zone chaude. CNESST.

[11] (2021). SRAS-CoV-2 : Mesures de prévention, de contrôle et de gestion des éclosions pour tous les milieux de soins. Institut National de Santé Publique du Québec.

[12] Hoernke K, Djellouli N, Andrews L, Lewis-Jackson S, Manby L, Martin S, Vanderslott S, Vindrola-Padros C (2021). Frontline healthcare workers' experiences with personal protective equipment during the COVID-19 pandemic in the UK: a rapid qualitative appraisal. BMJ Open.

[13] Chu DK, Akl EA, Duda S, Solo K, Yaacoub S, Schünemann HJ, COVID-19 Systematic Urgent Review Group Effort (SURGE) study authors (2020). Physical distancing, face masks, and eye protection to prevent person-to-person transmission of SARS-CoV-2 and COVID-19: a systematic review and meta-analysis. Lancet.

[14] Rebmann T, Vassallo A, Holdsworth JE (2021). Availability of personal protective equipment and infection prevention supplies during the first month of the COVID-19 pandemic: a national study by the APIC COVID-19 task force. Am J Infect Control.

[15] Jain U (2020). Risk of COVID-19 due to shortage of personal protective equipment. Cureus.

[16] Turer RW, Jones I, Rosenbloom ST, Slovis C, Ward MJ (2020). Electronic personal protective equipment: a strategy to protect emergency department providers in the age of COVID-19. J Am Med Inform Assoc.

[17] Lavoie B, Bourque CJ, Côté AJ, Rajagopal M, Clerc P, Bourdeau V, Ali S, Doyon-Trottier E, Castonguay V, Fontaine-Pagé É, Burstein B, Desaulniers P, Goldman RD, Thompson G, Berthelot S, Lagacé M, Gaucher N (2022). The responsibility to care: lessons learned from emergency department workers' perspectives during the first wave of the COVID-19 pandemic in Canada. CJEM.

[18] Tian Z, Stedman M, Whyte M, Anderson SG, Thomson G, Heald A (2020). Personal protective equipment (PPE) and infection among healthcare workers - what is the evidence?. Int J Clin Pract.

[19] Gross JV, Mohren J, Erren TC (2021). COVID-19 and healthcare workers: a rapid systematic review into risks and preventive measures. BMJ Open.

[20] Notarte KI, Catahay JA, Velasco JV, Pastrana A, Ver AT, Pangilinan FC, Peligro PJ, Casimiro M, Guerrero JJ, Gellaco MML, Lippi G, Henry BM, Fernández-de-Las-Peñas C (2022). Impact of COVID-19 vaccination on the risk of developing long-COVID and on existing long-COVID symptoms: a systematic review. EClinicalMedicine.

[21] Rezapour A, Souresrafil A, Peighambari MM, Heidarali M, Tashakori-Miyanroudi M (2021). Economic evaluation of programs against COVID-19: a systematic review. Int J Surg.

[22] Risko N, Werner K, Offorjebe OA, Vecino-Ortiz AI, Wallis LA, Razzak J (2020). Cost-effectiveness and return on investment of protecting health workers in low- and middle-income countries during the COVID-19 pandemic. PLoS One.

[23] Savitsky LM, Albright CM (2020). Preventing COVID-19 transmission on labor and delivery: a decision analysis. Am J Perinatol.

[24] Prata JC, Silva ALP, Walker TR, Duarte AC, Rocha-Santos T (2020). COVID-19 pandemic repercussions on the use and management of plastics. Environ Sci Technol.

[25] Zhang EJ, Aitchison LP, Phillips N, Shaban RZ, Kam AW (2021). Protecting the environment from plastic PPE. BMJ.

[26] Fang L, Pinder A, Cooper G, McGrath B, Shelton C (2021). Mitigating the environmental impact of plastic PPE: more than just disposal. BMJ.

[27] Rizan C, Reed M, Bhutta MF (2021). Environmental impact of personal protective equipment distributed for use by health and social care services in England in the first six months of the COVID-19 pandemic. J R Soc Med.

[28] Porter ME, Teisberg EO (2006). Redefining Health Care: Creating Value-Based Competition on Results.

[29] Porter ME (2009). A strategy for health care reform — toward a value-based system. N Engl J Med.

[30] Porter ME (2010). What is value in health care?. N Engl J Med.

[31] Alderwick H, Robertson R, Appleby J, Dunn P, Maguire D (2015). Better value in the NHS: the role of changes in clinical practice. The King’s Fund.

[32] (2016). Value-based healthcare in Europe: laying the foundation. The Economist Intelligence Unit.

[33] (2018). Value-based healthcare summit: transforming healthcare by redefining value. Canadian Foundation for Healthcare Improvement.

[34] Yount KW, Turrentine FE, Lau CL, Jones RS (2015). Putting the value framework to work in surgery. J Am Coll Surg.

[35] Wylie K, Crilly J, Toloo GS, FitzGerald G, Burke J, Williams G, Bell A (2015). Review article: emergency department models of care in the context of care quality and cost: a systematic review. Emerg Med Australas.

[36] (2021). Environmental management — eco-efficiency assessment of product systems — principles, requirements and guidelines. International Organization for Standardization.

[37] (2006). Eco-efficiency learning module. World Business Council for Sustainable Development.

[38] (2008). Indicateurs existants en faveur d'une production durable: constatations préliminaires. Organisation de coopération et de développement économiques.

[39] Page MJ, McKenzie JE, Bossuyt PM, Boutron I, Hoffmann TC, Mulrow CD, Shamseer L, Tetzlaff JM, Akl EA, Brennan SE, Chou R, Glanville J, Grimshaw JM, Hróbjartsson A, Lalu MM, Li T, Loder EW, Mayo-Wilson E, McDonald S, McGuinness LA, Stewart LA, Thomas J, Tricco AC, Welch VA, Whiting P, Moher D (2021). The PRISMA 2020 statement: an updated guideline for reporting systematic reviews. BMJ.

[40] Higgins JPT, Altman DG, Gøtzsche PC, Jüni P, Moher D, Oxman AD, Savovic J, Schulz KF, Weeks L, Sterne JAC (2011). The cochrane collaboration's tool for assessing risk of bias in randomised trials. BMJ.

[41] Sterne JA, Hernán MA, Reeves BC, Savović J, Berkman ND, Viswanathan M, Henry D, Altman DG, Ansari MT, Boutron I, Carpenter JR, Chan AW, Churchill R, Deeks JJ, Hróbjartsson A, Kirkham J, Jüni P, Loke YK, Pigott TD, Ramsay CR, Regidor D, Rothstein HR, Sandhu L, Santaguida PL, Schünemann HJ, Shea B, Shrier I, Tugwell P, Turner L, Valentine JC, Waddington H, Waters E, Wells GA, Whiting PF, Higgins JP (2016). ROBINS-I: a tool for assessing risk of bias in non-randomised studies of interventions. BMJ.

[42] Bun RS, Scheer J, Guillo S, Tubach F, Dechartres A (2020). Meta-analyses frequently pooled different study types together: a meta-epidemiological study. J Clin Epidemiol.

[43] Reeves BC, Deeks JJ, Higgins JPT, Shea B, Tugwell P, Wells GA, Chandler J, Thomas J, Higgins JPT, Page MJ, Cumpston M, Li T, Welch VA, Cochrane Non-Randomized Studies of Interventions Methods Group (2021). Chapter 24: Including non-randomized studies on intervention effects. Cochrane Handbook for Systematic Reviews of Interventions Version 6.2.

[44] Deeks JJ, Higgins JPT, Altman DG, Thomas J, Chandler J, Cumpston M, Li T, Page M, Welch V, Cochrane Statistical Methods Group (2021). Chapter 10: Analysing data and undertaking meta-analyses. Cochrane Handbook for Systematic Reviews of Interventions Version 6.2.

[45] Schünemann H, Higgins JPT, Vist G, Glasziou P, Akl E, Skoetz N, Guyatt G, Higgins JPT, Thomas J, Chandler J, Cumpston M, Li T, Page M, Welch V (2021). Chapter 14: Completing 'summary of findings' tables and grading the certainty of the evidence. Cochrane Handbook for Systematic Reviews of Interventions Version 6.2.

[46] Kaplan RS (2014). Improving value with TDABC. Healthc Financ Manage.

[47] Kaplan RS, Anderson SR (2004). Time-driven activity-based costing. Harv Bus Rev.

[48] Öker F, Özyapıcı H (2013). A new costing model in hospital management: time-driven activity-based costing system. Health Care Manag (Frederick).

[49] Erhun F, Mistry B, Platchek T, Milstein A, Narayanan VG, Kaplan RS (2015). Time-driven activity-based costing of multivessel coronary artery bypass grafting across national boundaries to identify improvement opportunities: study protocol. BMJ Open.

[50] Yun BJ, Prabhakar AM, Warsh J, Kaplan R, Brennan J, Dempsey KE, Raja AS (2016). Time-driven activity-based costing in emergency medicine. Ann Emerg Med.

[51] Kirkpatrick JR, Marks S, Slane M, Kim D, Cohen L, Cortelli M, Plate J, Perryman R, Zapas J (2015). Using value-based analysis to influence outcomes in complex surgical systems. J Am Coll Surg.

[52] Demeere N, Stouthuysen K, Roodhooft F (2009). Time-driven activity-based costing in an outpatient clinic environment: development, relevance and managerial impact. Health Policy.

[53] Akhavan S, Ward L, Bozic KJ (2016). Time-driven activity-based costing more accurately reflects costs in arthroplasty surgery. Clin Orthop Relat Res.

[54] Tseng P, Kaplan RS, Richman BD, Shah MA, Schulman KA (2018). Administrative costs associated with physician billing and insurance-related activities at an academic health care system. JAMA.

[55] Liu Y, Luciani-Mcgillivray I, Hughes M, Raja AS, Kaplan RS, Yun BJ (2020). Time-driven activity-based costing of emergency department postdischarge nurse calls. J Healthc Manag.

[56] Anzai Y, Heilbrun ME, Haas D, Boi L, Moshre K, Minoshima S, Kaplan R, Lee VS (2017). Dissecting costs of CT study: application of TDABC (Time-driven Activity-based Costing) in a tertiary academic center. Acad Radiol.

[57] Berthelot S, Mallet M, Baril L, Vezo A, Bissonnette L, Dupont PP, Blais S, Létourneau M, Bécotte G, Côté S, Lafrenière M, Émond M, Stelfox HT, Moore L (2017). A time-driven activity-based costing method for value-based analysis in the emergency department. Acad Emerg Med.

[58] Berthelot S, Mallet M, Baril L, Dupont P, Bissonnette L, Stelfox H, Émond M, Blais S, Vezo A, Létourneau M, Côté S, Bécotte G, Lafrenière M, Moore L (2017). P017: A time-driven activity-based costing method to estimate health care costs in the emergency department. Can J Emerg Med.

[59] Berthelot S, Breton M, Guertin JR, Archambault PM, Pelletier EB, Blouin D, Borgundvaag B, Duhoux A, Labbé LH, Laberge M, Lachapelle P, Lapointe-Shaw L, Layani G, Lefebvre G, Mallet M, Matthews D, McBrien K, McLeod S, Mercier E, Messier A, Moore L, Morris J, Morris K, Ovens H, Pageau P, Paquette JS, Perry J, Schull M, Simon M, Simonyan D, Stelfox HT, Talbot D, Vaillancourt S (2021). A value-based comparison of the management of ambulatory respiratory diseases in walk-in clinics, primary care practices, and emergency departments: protocol for a multicenter prospective cohort study. JMIR Res Protoc.

[60] Berthelot S, Mallet M, Blais S, Moore L, Guertin JR, Boulet J, Boilard C, Fortier C, Huard B, Mokhtari A, Lesage A, Lévesque É, Baril L, Olivier P, Vachon K, Yip O, Bouchard M, Simonyan D, Létourneau M, Pineault A, Vézo A, Stelfox HT (2022). Adaptation of time-driven activity-based costing to the evaluation of the efficiency of ambulatory care provided in the emergency department. J Am Coll Emerg Physicians Open.

[61] Marx T, Moore L, Talbot D, Guertin JR, Lachapelle P, Blais S, Singbo N, Simonyan D, Lavallée J, Zada N, Shahrigharahkoshan S, Huard B, Olivier P, Mallet M, Létourneau M, Lafrenière M, Archambault PM, Berthelot S (2023). A value-based comparison of the management of respiratory diseases in walk-in clinics and emergency departments. CJEM.

[62] Eckelman MJ, Sherman JD, MacNeill AJ (2018). Life cycle environmental emissions and health damages from the Canadian healthcare system: an economic-environmental-epidemiological analysis. PLoS Med.

[63] Eckelman M, Mosher M, Gonzalez A, Sherman J (2012). Comparative life cycle assessment of disposable and reusable laryngeal mask airways. Anesth Analg.

[64] Thiel CL, Cassels-Brown A, Goel H, Stancliffe R, Steinbach I, Thomas P, Vendries J (2020). Utilizing off-the-shelf LCA methods to develop a ‘triple bottom line’ auditing tool for global cataract surgical services. Resour Conserv Recycl.

[65] (2006). ISO 14040:2006(fr) Management environnemental — analyse du cycle de vie — principes et cadre. Organisation Internationale de Normalisation.

[66] (2006). ISO 14044:2006(fr) Management environnemental — analyse du cycle de vie — exigences et lignes directrices. Organisation Internationale de Normalisation.

[67] Jolliet O, Saadé-Sbeih M, Crettaz P, Jolliet-Gavin N, Shaked S (2017). Analyse du Cycle de Vie: Comprendre et Réaliser un Ecobilan, 3e Édition.

[68] Martel S, Steensma C (2012). Les années de vie corrigées de l’incapacité : un indicateur pour évaluer le fardeau de la maladie au Québec. Institut National de Santé Publique du Québec.

[69] IMPACT World+.

[70] (2021). Données COVID-19 au Québec. Institut National de Santé Publique du Québec.

[71] Harbec N, Boulard S (2019). Déploiement du système de coûts par parcours de soins et services (CPSS). Ministère de la Santé et des Services sociaux.

[72] Pike J, Grosse SD (2018). Friction cost estimates of productivity costs in cost-of-illness studies in comparison with human capital estimates: a review. Appl Health Econ Health Policy.

[73] Wong LP (2008). Focus group discussion: a tool for health and medical research. Singapore Med J.

[74] Freeman T (2006). 'Best practice' in focus group research: making sense of different views. J Adv Nurs.

[75] Ruff CC, Alexander IM, McKie C (2005). The use of focus group methodology in health disparities research. Nurs Outlook.

[76] Head M, Magnan M, Kurz WA, Levasseur A, Beauregard R, Margni M (2021). Temporally-differentiated biogenic carbon accounting of wood building product life cycles. SN Appl Sci.

[77] Houssard C, Maxime D, Pouliot Y, Margni M (2021). Allocation is not enough! a system boundaries expansion approach to account for production and consumption synergies: the environmental footprint of Greek yogurt. J Clean Prod.

[78] Lonca G, Muggéo R, Tétreault-Imbeault H, Bernard S, Margni M, Benetto E, Gericke K, Guiton M (2018). A bi-dimensional assessment to measure the performance of circular economy: a case study of tires end-of-life management. Designing Sustainable Technologies, Products and Policies: From Science to Innovation.

[79] Middelhauve L, Santecchia A, Girardin L, Maréchal F, Margni M (2020). Key performance indicators for decision making in building energy systems. https://infoscience.epfl.ch/record/278614.

[80] Mikhaylin S, Patouillard L, Margni M, Bazinet L (2018). Milk protein production by a more environmentally sustainable process: bipolar membrane electrodialysis coupled with ultrafiltration. Green Chem.

[81] Moore L, Guertin JR, Tardif PA, Ivers NM, Hoch J, Conombo B, Antony J, Stelfox HT, Berthelot S, Archambault P, Turgeon A, Gandhi R, Grimshaw JM (2022). Economic evaluations of audit and feedback interventions: a systematic review. BMJ Qual Saf.

[82] Porgo TV, Moore L, Truchon C, Berthelot S, Stelfox HT, Cameron PA, Gabbe BJ, Hoch JS, Evans DC, Lauzier F, Bernard F, Turgeon AF, Clément J (2019). Patient-level resource use for injury admissions in Canada: a multicentre retrospective cohort study. Injury.

[83] Watts N, Amann M, Arnell N, Ayeb-Karlsson S, Belesova K, Boykoff M, Byass P, Cai W, Campbell-Lendrum D, Capstick S, Chambers J, Dalin C, Daly M, Dasandi N, Davies M, Drummond P, Dubrow R, Ebi KL, Eckelman M, Ekins P, Escobar LE, Montoya LF, Georgeson L, Graham H, Haggar P, Hamilton I, Hartinger S, Hess J, Kelman I, Kiesewetter G, Kjellstrom T, Kniveton D, Lemke B, Liu Y, Lott M, Lowe R, Sewe MO, Martinez-Urtaza J, Maslin M, McAllister L, McGushin A, Mikhaylov SJ, Milner J, Moradi-Lakeh M, Morrissey K, Murray K, Munzert S, Nilsson M, Neville T, Oreszczyn T, Owfi F, Pearman O, Pencheon D, Phung D, Pye S, Quinn R, Rabbaniha M, Robinson E, Rocklöv J, Semenza JC, Sherman J, Shumake-Guillemot J, Tabatabaei M, Taylor J, Trinanes J, Wilkinson P, Costello A, Gong P, Montgomery H (2019). The 2019 report of the lancet countdown on health and climate change: ensuring that the health of a child born today is not defined by a changing climate. Lancet.

[84] (2019). Le lancet countdown sur la santé et les changements climatiques 2019 : compte rendu à l'intention du Canada. Association Médicale Canadienne.

[85] Linstadt H, Collins A, Slutzman JE, Kimball E, Lemery J, Sorensen C, Winstead-Derlega C, Evans K, Auerbach PS (2020). The climate-smart emergency department: a primer. Ann Emerg Med.

