# Supplementary material for: Postpandemic Evaluation of the Eco-Efficiency of Personal Protective Equipment Against COVID-19 in Emergency Departments: Proposal for a Mixed Methods Study
Source: JMIR Res Protoc. 2023 Dec 7;12:e50682. doi: 10.2196/50682 (PMC10739239; doi:10.2196/50682)
Supplement: Multimedia Appendix 1 [file resprot_v12i1e50682_app1.pdf]

PRÉCAUTIONS
GOUTTELETTES/CONTACT
+ PROTECTION OCULAIRE

Visiteurs Se présenter au poste des infirmières avant d'entrer

À L'ENTRÉE

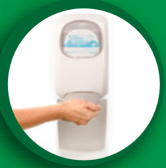

Pratiquer l'hygiène des mains

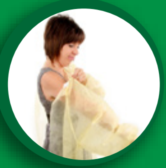

Revêtir la blouse

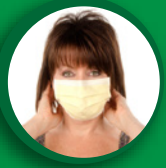

Porter le masque

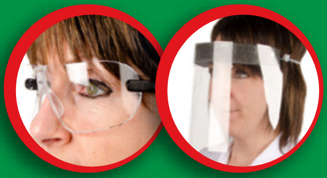

Porter la protection oculaire

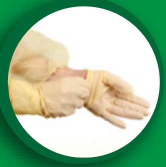

Enfiler les gants

À LA SORTIE

DANS LA CHAMBRE

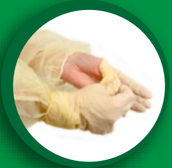

Retirer les gants

Pratiquer l'hygiène des mains

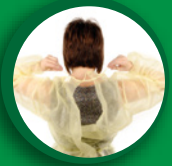

Retirer la blouse

Pratiquer l'hygiène des mains

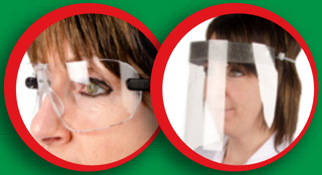

Retirer la protection oculaire

Pratiquer l'hygiène des mains

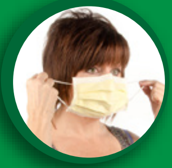

Retirer le masque de procédure dans la chambre AVANT de sortir

Pratiquer l'hygiène des mains

À L'EXTÉRIEUR DE LA CHAMBRE

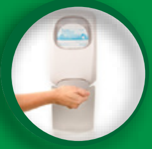

Pratiquer l'hygiène des mains

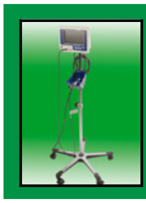

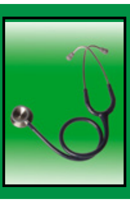

Matériel dédié ou désinfecté après usage

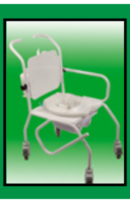

COMM 19_000_01-PCI

GRM 322865
