# Supplementary material for: Postpandemic Evaluation of the Eco-Efficiency of Personal Protective Equipment Against COVID-19 in Emergency Departments: Proposal for a Mixed Methods Study
Source: JMIR Res Protoc. 2023 Dec 7;12:e50682. doi: 10.2196/50682 (PMC10739239; doi:10.2196/50682)
Supplement: Multimedia Appendix 2 [file resprot_v12i1e50682_app2.pdf]

PRÉCAUTIONS AÉRIENNES/CONTACT RENFORCÉES

Visiteurs ACCÈS INTERDIT SANS AUTORISATION

À L'ENTRÉE

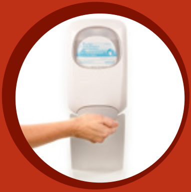

Pratiquer l'hygiène des mains

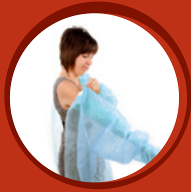

Revêtir la blouse hydrofuge

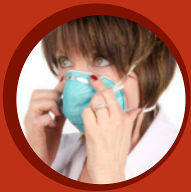

Porter le masque N-95
(Vérifier l'étanchéité)

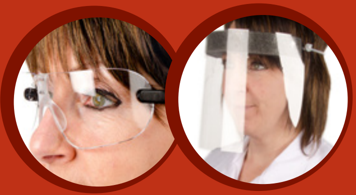

Porter la protection oculaire

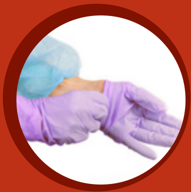

Enfiler les gants de nitrile

À LA SORTIE

DANS LA CHAMBRE

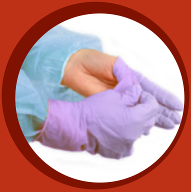

Retirer les gants
Pratiquer l'hygiène des mains

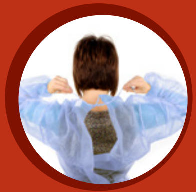

Retirer la blouse
Pratiquer l'hygiène des mains

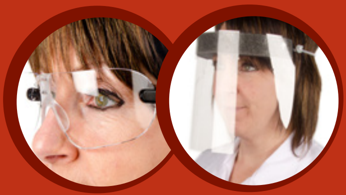

Retirer la protection oculaire

Pratiquer l'hygiène des mains

À L'EXTÉRIEUR DE LA CHAMBRE

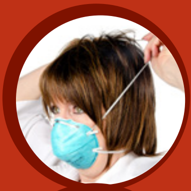

Pratiquer l'hygiène des mains
Retirer le masque N-95

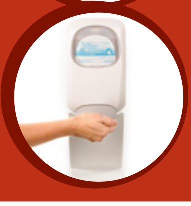

Pratiquer l'hygiène des mains

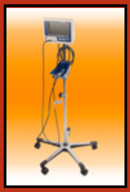

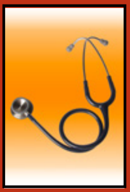

Chambre à pression négative
(Portes et fenêtres fermées)

Matériel dédié ou désinfecté après usage

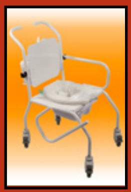

COMM 19_000_01-PCI

GRM 793459

Modifié 2020/09/15
