# Supplementary material for: Postpandemic Evaluation of the Eco-Efficiency of Personal Protective Equipment Against COVID-19 in Emergency Departments: Proposal for a Mixed Methods Study
Source: JMIR Res Protoc. 2023 Dec 7;12:e50682. doi: 10.2196/50682 (PMC10739239; doi:10.2196/50682)
Supplement: Multimedia Appendix 3 [file resprot_v12i1e50682_app3.pdf]

Medline (Ovid)
Date of the search: 22-03-2021
Database limit: No database limit has been apply

Concepts	#	Search strategy	Results
Personal Protective Equipment (Controlled vocabulary)	1	"Personal Protective Equipment/ OR exp "Eye Protective Devices/ OR exp Masks/ OR Protective Clothing/ OR "Gloves, Protective"/ OR "Respiratory Protective Devices"/ OR N95 Respirators/	20002
Personal Protective Equipment (Free vocabulary)	2	((protecti* or safety) adj2 (visor or eyeglass* or eye or lenses or device* or clothing or equipment or glasse* or suit or gloves)) or Goggle* or Mask* or gown or "Filtering Facepiece Respirator*" or (N95 adj2 (facemask* or filtering or mask* or respirator*)).ti,ab,kw	100250
Personal Protective Equipment (Combined)	3	or/1-2	110014
Coronavirus (Controlled vocabulary)	4	Coronaviridae/ OR Coronavirus/ OR Coronavirus Infections/ OR betacoronavirus/ OR COVID-19/ OR SARS-CoV-2/	74368
Coronavirus (Free vocabulary)	5	coronavirus.ti,ab,kw OR "corona virus".ti,ab,kw OR coronavirinae.ti,ab,kw OR coronaviridae.ti,ab,kw OR betacoronavirus.ti,ab,kw OR "SARS-CoV-2".ti,ab,kw OR covid19.ti,ab,kw OR "covid 19".ti,ab,kw OR 2019ncov.ti,ab,kw OR "2019-nCoV".ti,ab,kw OR "novel cov".ti,ab,kw	123353
Coronavirus (combined)	6	or/4-5	128870
Hospital outcomes (Controlled vocabulary)	7	Occupational Exposure/ OR Cross Infection/ OR "Disease Transmission, Infectious"/ OR Infectious Disease Transmission, Patient-to-Professional/ OR Infectious Disease Transmission, Professional-to-Patient/ OR Environmental Exposure/ OR Disease Outbreaks/ OR Contact Tracing/ OR Infection Control/ OR exp Hospitalization/ OR Absenteeism/ OR exp Mortality/	912358
Hospital outcomes (Free vocabulary)	8	Exposure*.ti,ab,kw OR Transmission.ti,ab,kw OR Infection.ti,ab,kw OR ((Patient OR hospital OR "intensive care") adj2 Admission).ti,ab,kw OR Mortality.ti,ab,kw OR "Death Rate*".ti,ab,kw OR ((work OR job) adj2 (absence OR Absenteeism)).ti,ab,kw	3017057
Hospital outcomes (combined)	9	or/7-8	3555462
Review search filter	10	Systematic Review/ OR Review/ OR Meta-Analysis/ OR meta-analysis as topic/ OR "Review Literature as Topic"/ OR "systematic review (topic)"/ OR ((literature* OR integrative OR map* OR narrative* OR "State-of-the-art" OR rapid? OR realist? OR systemati* OR umbrella*) adj2 review*).ti,ab,kw OR (scoping adj2 (stud* OR review)).ti,ab,kw OR (evidence adj2 map*).ti,ab,kw OR (("mixed stud*" OR "mixed method*" OR "meta-narrative*") adj3 (review* or synthes*)).ti,ab,kw OR ("Environmental scan*" OR "systematic map*" OR "evidence synthesis*" OR "meta-ethnograph*" OR "review of reviews").ti,ab,kw	3005336
Combination of concepts	11	(3 AND 6 AND 9) NOT 10	2732
Studies from december 2019 only	12	limit 11 to dt=20191201-20210322	2665

Embase (Embase.com)
Date of the search: 22-03-2021
Database limit: Embase results only limit has been apply

Concepts	#	Search strategy	Results
Personal Protective Equipment (Controlled vocabulary)	1	'protective equipment'/de OR 'eye protective device'/exp OR 'protective glasses'/exp OR 'protective clothing'/exp OR 'mask'/exp OR 'filtering facepiece respirator'/exp OR 'protective glove'/exp OR 'latex glove'/de OR 'respiratory protection'/exp	75,221
Personal Protective Equipment (Free vocabulary)	2	((protecti* OR safety) NEAR/2 (visor OR eyeglass* OR eye OR lenses OR device* OR clothing OR equipment OR glasse* OR suit OR gloves)):ti,ab,kw OR Goggle*:ti,ab,kw OR Mask*:ti,ab,kw OR gown:ti,ab,kw OR "Filtering Facepiece Respirator*":ti,ab,kw OR (N95 NEAR/2 (facemask* OR filtering OR mask* OR respirator*)):ti,ab,kw	127,489
Personal Protective Equipment (Combined)	3	#1 OR #2	176,386
Coronavirus (Controlled vocabulary)	4	'Coronaviridae'/de OR 'Coronavirus infection'/de OR 'coronavirus disease 2019'/de OR 'Betacoronavirus'/exp OR 'SARS-related coronavirus'/exp	119,144
Coronavirus (Free vocabulary)	5	coronavirus:ti,ab,kw OR 'corona virus':ti,ab,kw OR coronavirinae:ti,ab,kw OR coronaviridae:ti,ab,kw OR betacoronavirus:ti,ab,kw OR 'sars-cov-2':ti,ab,kw OR covid19:ti,ab,kw OR 'covid 19':ti,ab,kw OR 2019ncov:ti,ab,kw OR '2019-ncov':ti,ab,kw OR 'novel cov':ti,ab,kw	121,319
Coronavirus (combined)	6	#4 OR #5	135,085
Hospital outcomes (Controlled vocabulary)	7	'occupational exposure'/de OR 'cross infection'/de OR 'disease transmission'/exp OR 'horizontal disease transmission'/exp OR 'nosocomial transmission'/exp OR 'environmental exposure'/exp OR 'epidemic'/de OR 'contact examination'/de OR 'infection control'/de OR 'hospitalization'/de OR 'mortality'/de OR 'hospital mortality'/de OR 'mortality rate'/exp OR 'hospital admission'/de OR 'absenteeism'/de	1,931,711
Hospital outcomes (Free vocabulary)	8	Exposure*:ti,ab,kw OR Transmission:ti,ab,kw OR Infection:ti,ab,kw OR ((Patient or hospital OR "intensive care") NEAR/2 Admission):ti,ab,kw OR Mortality:ti,ab,kw OR "Death Rate*":ti,ab,kw OR ((work OR job) NEAR/2 (absence OR Absenteeism)):ti,ab,kw	4,004,086
Hospital outcomes (combined)	9	#7 OR #8	4,869,632
Review search filter	10	'review'/exp OR 'meta analysis'/exp OR 'systematic review (topic)'/de OR 'meta analysis (topic)'/de OR ((literature* OR integrative OR map* OR narrative* OR "State-of-the-art" OR rapid$ OR realist$ OR systemati* OR umbrella*) NEAR/2 review*):ti,ab,kw OR (scoping NEAR/2 (stud* OR review)):ti,ab,kw OR (evidence NEAR/2 map*):ti,ab,kw OR (("mixed stud*" OR "mixed method*" OR "meta-narrative*") NEAR/3 (review* or synthes*)):ti,ab,kw OR ("Environmental scan*" OR "systematic map*" OR "evidence synthesis*" OR "meta-ethnograph*" OR "review of reviews"):ti,ab,kw	3,090,951
Combination of concepts	11	(#3 AND #6 AND #9) NOT #10	6,295
Studies from december 2019 only	12	#11 AND [01-12-2019]/sd	6,132
Embase results only	13	#12 AND [embase]/lim NOT ([embase]/lim AND [medline]/lim)	1,526

CINAHL

Date of the search: 22-03-2021

Database limit: No database limit has been apply

Concepts	#	Search strategy	Results
Personal Protective Equipment (Controlled vocabulary)	1	MH "Personal Protective Equipment" OR MH Masks OR MH "Protective Clothing+" OR MH "Respiratory Protective Devices" OR MH "Eye Protective Devices" OR MH Gloves	13,610
Personal Protective Equipment (Free vocabulary)	2	TI ((protecti* OR safety) N2 (visor OR eyeglass* OR eye OR lenses OR device* OR clothing OR equipment OR glasse* OR suit OR gloves) OR Goggle* OR Mask* OR gown OR "Filtering Facepiece Respirator*" OR (N95 N2 (facemask* OR filtering OR mask* OR respirator*)) OR AB ((protecti* OR safety) N2 (visor OR eyeglass* OR eye OR lenses OR device* OR clothing OR equipment OR glasse* OR suit OR gloves) OR Goggle* OR Mask* OR gown OR "Filtering Facepiece Respirator*" OR (N95 N2 (facemask* OR filtering OR mask* OR respirator*))	28,054
Personal Protective Equipment (Combined)	3	S1 OR S2	36,321
Coronavirus (Controlled vocabulary)	4	MH "Coronavirus Infections" OR MH "COVID-19"	23,606
Coronavirus (Free vocabulary)	5	TI coronavirus OR AB coronavirus OR TI "corona virus" OR AB "corona virus" OR TI coronavirinae OR AB coronavirinae OR TI coronaviridae OR AB coronaviridae OR TI betacoronavirus OR AB betacoronavirus OR TI "SARS-CoV-2" OR AB "SARS-CoV-2" OR TI covid19 OR AB covid19 OR TI "covid 19" OR AB "covid 19" OR TI 2019ncov OR AB 2019ncov OR TI "2019-nCoV" OR AB "2019-nCoV" OR TI "novel cov" OR AB "novel cov"	28,418
Coronavirus (combined)	6	S4 OR S5	36,912
Hospital outcomes (Controlled vocabulary)	7	MH "Occupational Exposure" OR MH "Environmental Exposure" OR MH "Cross Infection" OR MH "Disease Transmission, Horizontal+" OR MH "Infection Control" OR MH "Contact Tracing" OR MH "Disease Outbreaks" OR MH Hospitalization OR MH "Patient Admission" OR MH Absenteeism OR MH "Mortality+"	249,172
Hospital outcomes (Free vocabulary)	8	TI Exposure* OR AB Exposure* OR TI Transmission OR AB Transmission OR TI Infection OR AB Infection OR TI ((Patient or hospital OR "intensive care") N2 Admission) OR AB ((Patient or hospital OR "intensive care") N2 Admission) OR TI Mortality OR AB Mortality OR TI "Death Rate*" OR AB "Death Rate*" OR TI ((work OR job) N2 ((absence OR Absenteeism)) OR AB ((work OR job) N2 ((absence OR Absenteeism))	565,565
Hospital outcomes (combined)	9	S7 OR S8	702,241
Review search filter	10	TI ((literature* OR integrative OR map* OR narrative* OR "State-of-the-art" OR rapid? OR realist? OR systemati* OR umbrella*) N2 review*) OR AB ((literature* OR integrative OR map* OR narrative* OR "State-of-the-art" OR rapid? OR realist? OR systemati* OR umbrella*) N2 review*) OR TI (scoping N2 (stud* OR review)) OR AB (scoping N2 (stud* OR review)) OR TI (evidence N2 map*) OR AB (evidence N2 map*) OR TI (("mixed stud*" OR "mixed method*" OR "meta-narrative*") N3 (review* or synthes*)) OR TI ("Environmental scan*" OR "systematic map*" OR "evidence synthesis*" OR "meta-ethnograph*" OR "review of reviews") OR AB ("Environmental scan*"	202,036

		OR "systematic map*" OR "evidence synthesis*" OR "meta-ethnograph*" OR "review of reviews")	
Combination of concepts	11	(S3 AND S6 AND S9) NOT S10	2,042
Studies from december 2019 only	12	S11 AND DT 20191201-20210322	1,995

Cochrane Library

Date of the search: 22-03-2021

Database limit: trials from december 2019 limits have been apply

Concepts	#	Search strategy	Results
Personal Protective Equipment (Controlled vocabulary)	1	[mh ^"Personal Protective Equipment"] OR [mh "Eye Protective Devices"] OR [mh "Masks"] OR [mh ^"Protective Clothing"] OR [mh ^"Gloves, Protective"] OR [mh "Respiratory Protective Devices"] OR [mh "N95 Respirators"]	2014
Personal Protective Equipment (Free vocabulary)	2	((protecti* OR safety) NEAR/2 (visor OR eyeglass* OR eye OR lenses OR device* OR clothing OR equipment OR glasse* OR suit OR gloves):ti,ab,kw OR Goggle*:ti,ab,kw OR Mask*:ti,ab,kw OR gown:ti,ab,kw OR "Filtering Facepiece Respirator*":ti,ab,kw OR (N95 NEAR/2 (facemask* OR filtering OR mask* OR respirator*)):ti,ab,kw	26462
Personal Protective Equipment (Combined)	3	#1 OR #2	26462
Coronavirus (Controlled vocabulary)	4	[mh ^"COVID-19"] OR [mh ^"SARS-CoV-2"] OR [mh ^"Coronavirus Infections"] OR [mh ^Betacoronavirus]	710
Coronavirus (Free vocabulary)	5	coronavirus:ti,ab,kw OR "corona virus":ti,ab,kw OR coronavirinae:ti,ab,kw OR coronaviridae:ti,ab,kw OR betacoronavirus:ti,ab,kw OR "SARS-CoV-2":ti,ab,kw OR covid19:ti,ab,kw OR "covid 19":ti,ab,kw OR 2019ncov:ti,ab,kw OR "2019-nCoV":ti,ab,kw OR "novel cov":ti,ab,kw	4471
Coronavirus (combined)	6	#4 OR #5	4471
Hospital outcomes (Controlled vocabulary)	7	[mh ^"Occupational Exposure"] OR [mh ^"Cross Infection"] OR [mh ^"Disease Transmission, Infectious"] OR [mh ^"Infectious Disease Transmission, Patient-to-Professional"] OR [mh ^"Infectious Disease Transmission, Professional-to-Patient"] OR [mh ^"Environmental Exposure"] OR [mh ^"Disease Outbreaks"] OR [mh ^"Contact Tracing"] OR [mh ^"Infection Control"] OR [mh Hospitalization] OR [mh ^Absenteeism] OR [mh Mortality]	29641
Hospital outcomes (Free vocabulary)	8	Exposure*:ti,ab,kw OR Transmission:ti,ab,kw OR Infection:ti,ab,kw OR ((Patient OR hospital OR "intensive care") NEAR/2 Admission):ti,ab,kw OR Mortality:ti,ab,kw OR "Death Rate*":ti,ab,kw OR ((work OR job) NEAR/2 (absence OR Absenteeism)):ti,ab,kw	230817
Hospital outcomes (combined)	9	#7 OR #8	241891
Combination of concepts	10	(#3 AND #6 AND #9)	255
Clinical trials studies only	11	n/a	247
Clinical trials from december 2019 only	12	n/a	241

Web of science
Date of the search: 22-03-2021
Database limit: Studies from 2019 limit has been apply

Concepts	#	Search strategy	Results
Personal Protective Equipment (Combined)	1	TS=((protecti* OR safety) NEAR/2 (visor OR eyeglass* OR eye OR lenses OR device* OR clothing OR equipment OR glasse* OR suit OR gloves)) OR TS=Goggle* OR TS=Mask* OR TS=gown OR TS="Filtering Facepiece Respirator*" OR TS=(N95 NEAR/2 (facemask* OR filtering OR mask* OR respirator*))	29,279
Coronavirus (combined)	2	TS=(coronavirus OR "corona virus" OR coronavirinae OR coronaviridae OR betacoronavirus OR "SARS-CoV-2" OR covid19 OR "covid 19" OR 2019ncov OR "2019-nCoV" OR "novel cov")	104,832
Hospital outcomes (combined)	3	TS=(Exposure* OR Transmission OR Infection) OR TS=((Patient or hospital OR "intensive care") NEAR/2 Admission) OR TS=Mortality OR TS="Death Rate*" OR TS=((work OR job) NEAR/2 (absence OR Absenteeism))	691,358
Review search filter	4	TS=((literature* OR integrative OR map* OR narrative* OR "State-of-the-art" OR rapid$ OR realist$ OR systemati* OR umbrella*) NEAR/2 review*) OR TS=(scoping NEAR/2 (stud* OR review)) OR TS=(evidence NEAR/2 map*) OR TS=(("mixed stud*" OR "mixed method*" OR "meta-narrative*") NEAR/3 (review* or synthes*)) OR TS=("Environmental scan*" OR "systematic map*" OR "evidence synthesis*" OR "meta-ethnograph*" OR "review of reviews")	154,808
Combination of concepts	5	(#1 AND #2 AND #3) NOT #4	2355

Epistemonikos (www.epistemonikos.org)
Date of the search: 22-03-2021
Database limit: Search in title/abstract only and primary studies from 2019 limits have been apply

Search strategy	Total number of results
("Personal Protective Device*" OR "Personal Protective Equipment" OR "Personal Safety Devices" OR "eye protecti*" OR "Safety Lenses" OR Goggles OR "Safety Glasses" OR Mask* OR "Protective Clothing" OR "Protective suit" OR "Protective Gloves" OR "Respirator* Protecti* Device*" OR "N95 Respirator*" OR "N95 Face Mask*" OR "N95 Mask*" OR "Filtering Facepiece Respirators") AND (coronavirus OR "corona virus" OR "coronavirinae" OR "coronaviridae" OR "betacoronavirus" OR "SARS-CoV-2" OR "covid19" OR "covid 19" OR "2019ncov" OR "2019-nCoV" OR "novel cov") AND (Exposure* OR Transmission OR Infection OR "Patient Admission" OR "Hospital admission" OR Mortality OR "Death Rate*" OR "admission to intensive care" OR "intensive care admission" OR "work Absenteeism" OR "job Absenteeism" OR "work absence" OR "job absence")	1939

ClinicalTrials.gov
Date of the search: 22-03-2021
Database limit: search coronavirus concept in disease field and Personal Protective Device concept in intervention field.

#	Search strategy	Results
1	(coronavirus OR "corona virus" OR "coronavirinae" OR "coronaviridae" OR "betacoronavirus" OR "SARS-CoV-2" OR "covid19" OR "covid 19" OR "2019ncov" OR "2019-nCoV" OR "novel cov") AND ("Personal Protective Device*" OR "Personal Protective Equipment" OR "Personal Safety Devices")	34
2	(coronavirus OR "corona virus" OR "coronavirinae" OR "coronaviridae" OR "betacoronavirus" OR "SARS-CoV-2" OR "covid19" OR "covid 19" OR "2019ncov" OR "2019-nCoV" OR "novel cov") AND ("eye protecti*" OR "Safety Lenses" OR Goggles OR "Safety Glasses" OR Mask*)	1

3	(coronavirus OR "corona virus" OR "coronavirinae" OR "coronaviridae" OR "betacoronavirus" OR "SARS-CoV-2" OR "covid19" OR "covid 19" OR "2019ncov" OR "2019-nCoV" OR "novel cov") AND ("Protective Clothing" OR "Protective suit" OR "Protective Gloves" OR "Respirator Protection Device" OR "Respirator Protective Device" OR "N95 Respirator*" OR "N95 Face Mask*" OR "N95 Mask*" OR "Filtering Facepiece Respirators")	8
	Total number of results	43

MedRxiv (https://www.medrxiv.org/)

Date of the search: 22-03-2021

Database limit: studies from 01 Dec, 2019, and MedRxiv database limits have been apply

#	Search strategy	Results
1	"covid-19" AND Mask AND Transmission	116
2	"covid-19" AND "Personal Protective Device" AND Transmission	5
3	"covid-19" AND "Personal Protective Equipment" AND Transmission	185
4	"covid-19" AND "N95 Respirator" AND Transmission	26
5	"SARS-CoV-2" AND Mask AND Transmission	73
6	"SARS-CoV-2" AND "Personal Protective Device" AND Transmission	3
7	"SARS-CoV-2" AND "Personal Protective Equipment" AND Transmission	86
8	"SARS-CoV-2" AND "N95 Respirator" AND Transmission	22
9	coronavirus AND Mask AND Transmission	35
10	coronavirus AND "Personal Protective Device" AND Transmission	2
11	coronavirus AND "Personal Protective Equipment" AND Transmission	58
12	coronavirus AND "N95 Respirator" AND Transmission	8
	Total number of results	619
