# Supplementary material for: Postpandemic Evaluation of the Eco-Efficiency of Personal Protective Equipment Against COVID-19 in Emergency Departments: Proposal for a Mixed Methods Study
Source: JMIR Res Protoc. 2023 Dec 7;12:e50682. doi: 10.2196/50682 (PMC10739239; doi:10.2196/50682)

Syndrome d'allure grippale parcours à l'urgence du CHUL

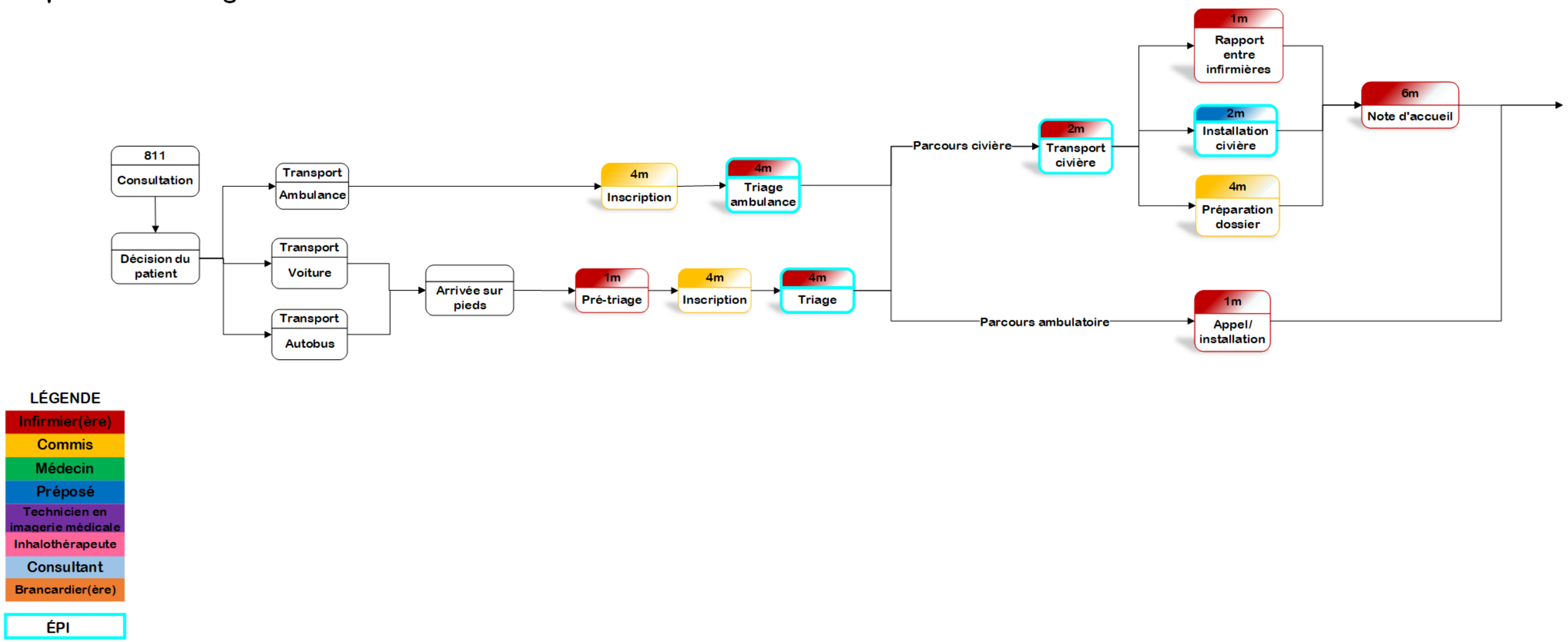

ÉPI = port d'un équipement de protection individuelle

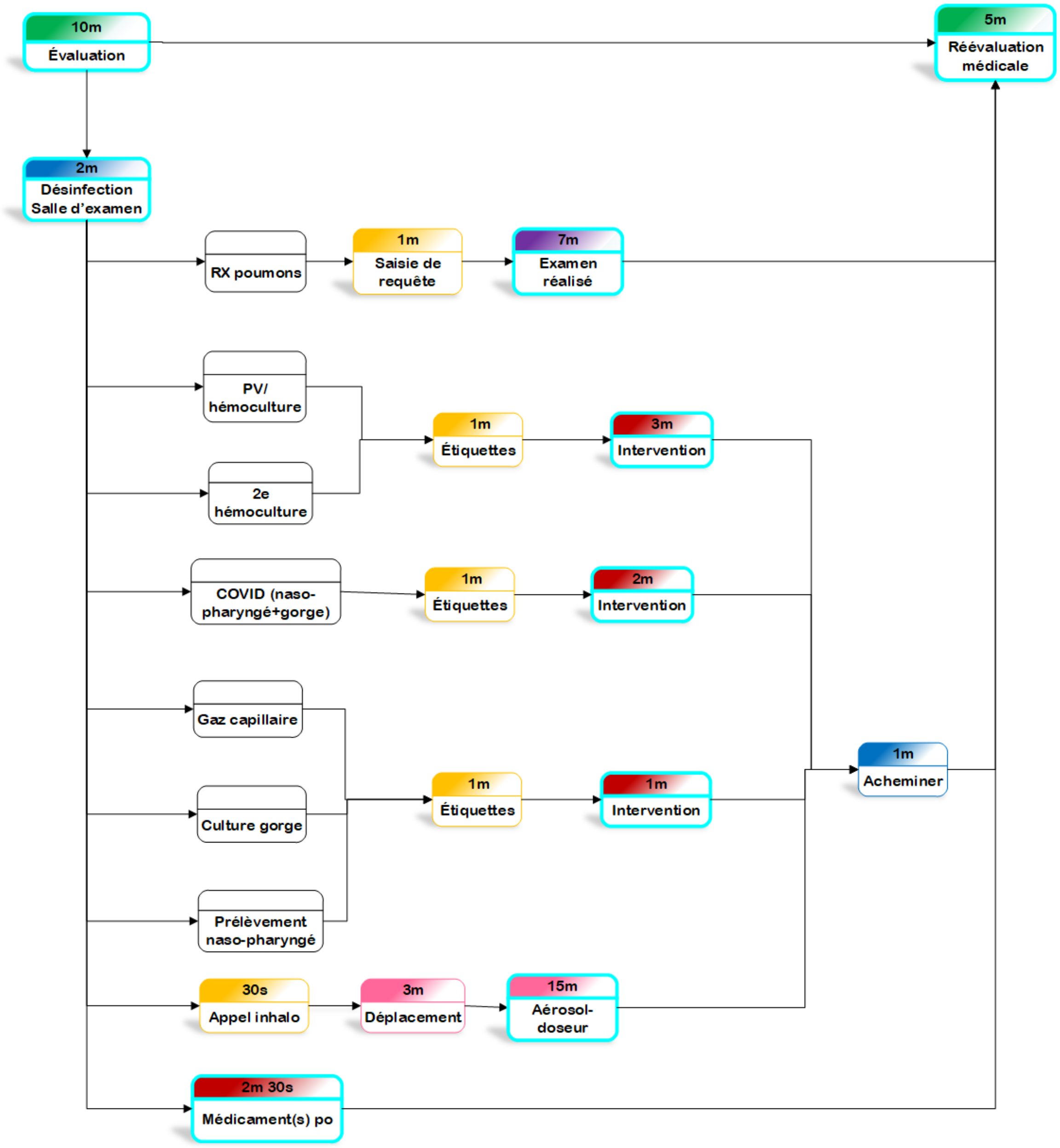
10m
Évaluation
5m
Réévaluation médicale
2m
Désinfection Salle d'examen
RX poumons
1m
Saisie de requête
7m
Examen réalisé
PV/ hémoculture
2e hémoculture
1m
Étiquettes
3m
Intervention
COVID (naso-pharyngé+gorge)
1m
Étiquettes
2m
Intervention
Gaz capillaire
Culture gorge
Prélèvement naso-pharyngé
1m
Étiquettes
1m
Intervention
1m
Acheminer
30s
Appel inhalo
3m
Déplacement
15m
Aérosol-doseur
2m 30s
Médicament(s) po

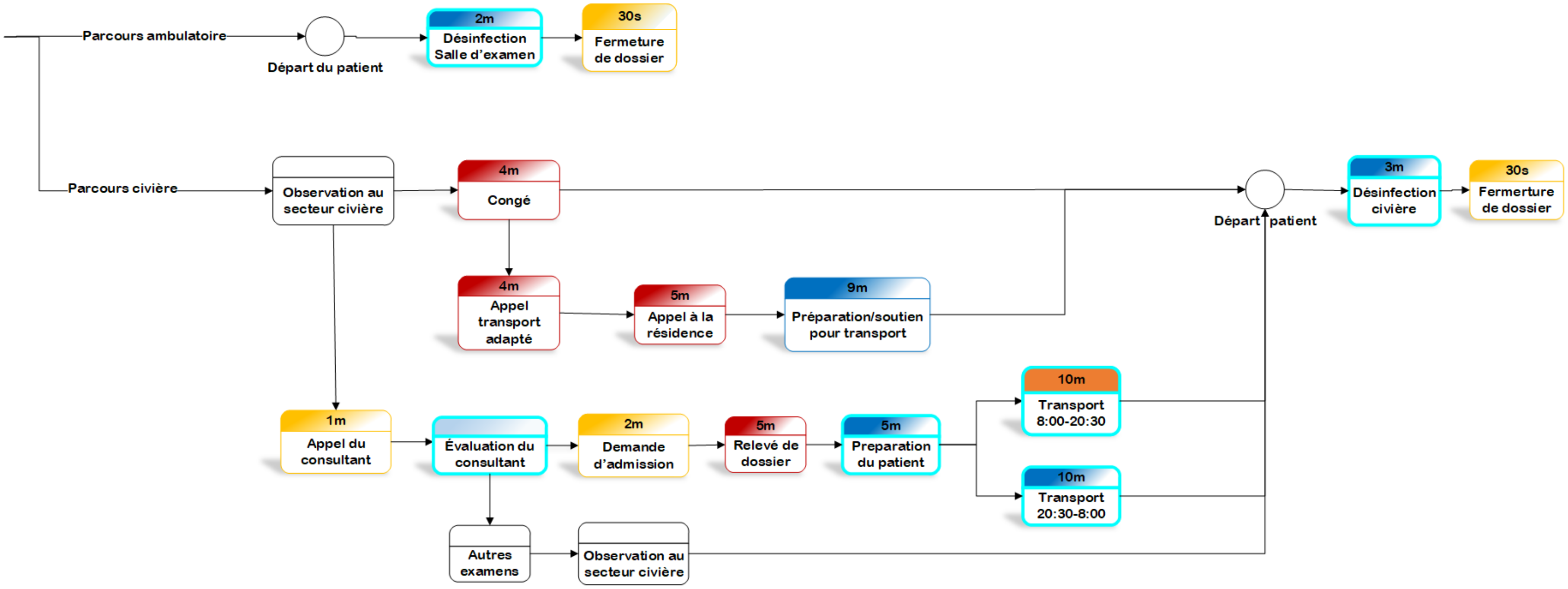
Parcours ambulatoire
Départ du patient
2m
Désinfection Salle d'examen
30s
Fermeture de dossier
Parcours civière
Observation au secteur civière
4m
Congé
Départ patient
3m
Désinfection civière
30s
Fermerture de dossier
4m
Appel transport adapté
5m
Appel à la résidence
9m
Préparation/soutien pour transport
1m
Appel du consultant
Évaluation du consultant
2m
Demande d'admission
5m
Relevé de dossier
5m
Preparation du patient
10m
Transport 8:00-20:30
10m
Transport 20:30-8:00
Autres examens
Observation au secteur civière

Syndrome d’allure grippale
parcours à l’urgence du CHUL

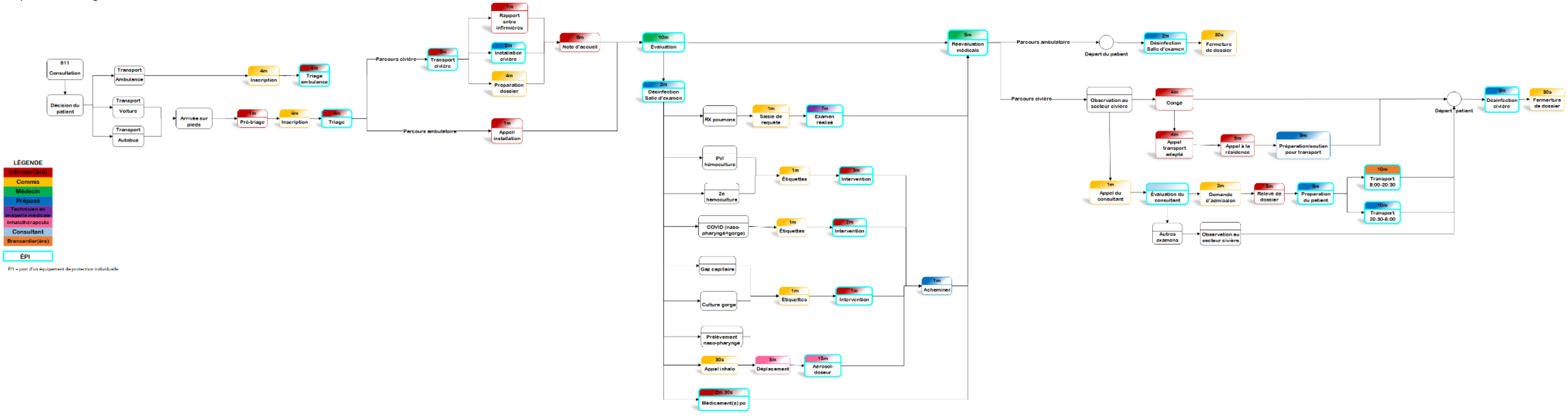

Supplement: Multimedia Appendix 4 [file resprot_v12i1e50682_app4.pdf]
