# Supplementary figures and images for: Postpandemic Evaluation of the Eco-Efficiency of Personal Protective Equipment Against COVID-19 in Emergency Departments: Proposal for a Mixed Methods Study
Source: JMIR Res Protoc. 2023 Dec 7;12:e50682. doi: 10.2196/50682 (PMC10739239; doi:10.2196/50682)

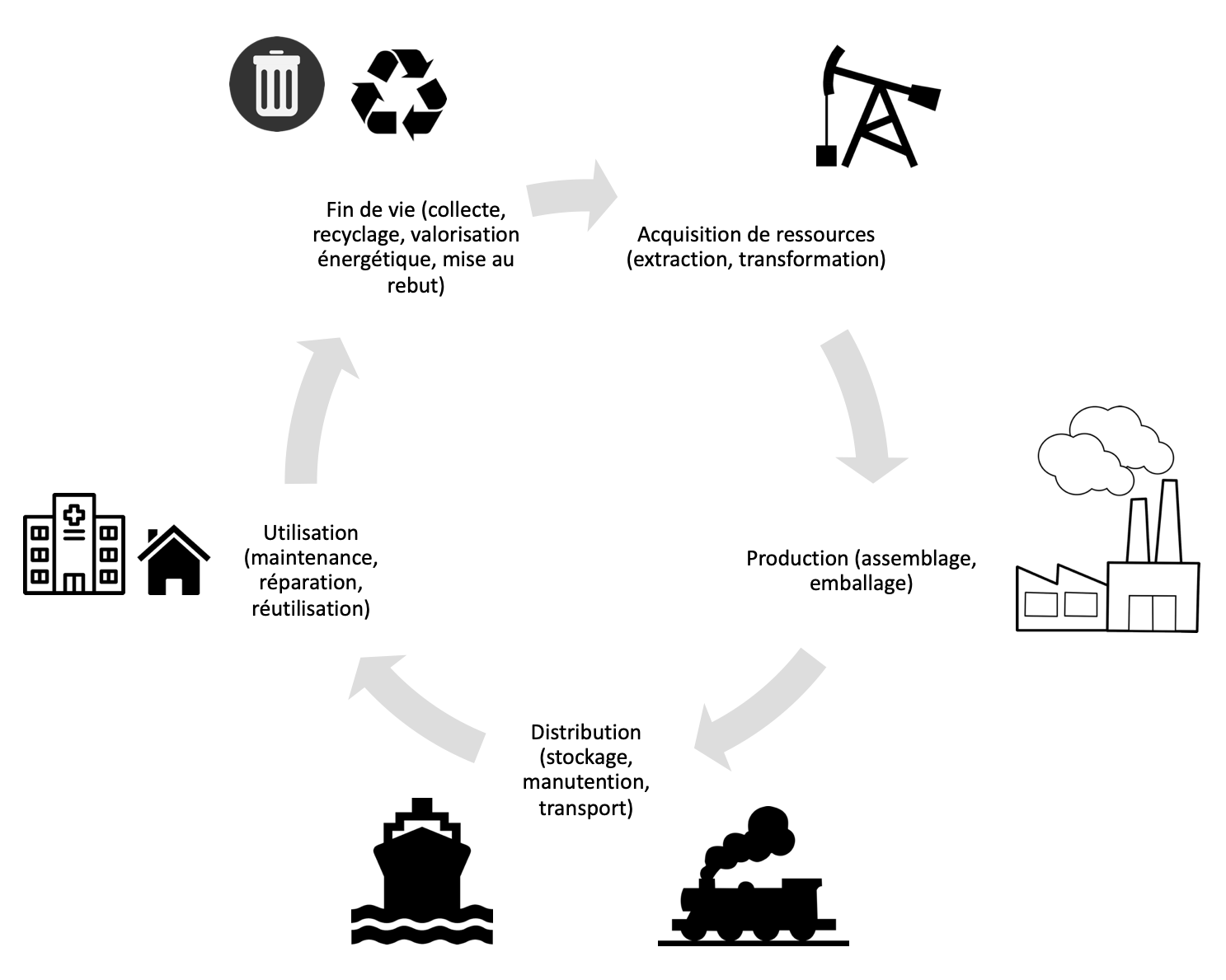

Supplement: Multimedia Appendix 5 [file resprot_v12i1e50682_app5.png]
