# Supplementary material for: Postpandemic Evaluation of the Eco-Efficiency of Personal Protective Equipment Against COVID-19 in Emergency Departments: Proposal for a Mixed Methods Study
Source: JMIR Res Protoc. 2023 Dec 7;12:e50682. doi: 10.2196/50682 (PMC10739239; doi:10.2196/50682)
Supplement: Multimedia Appendix 6 [file resprot_v12i1e50682_app6.pdf]

July/Juillet 15, 2021
DCO150GP

Canadian Institutes of Health Research/Instituts de recherche en santé du Canada

Notice of Recommendation/Avis de recommandation

Application Number/Numéro de la demande: 462760
Committee Code/Code du comité: PH3

Applicants/Candidats: Docteur Simon Berthelot; Docteur Yves Longtin; Prof. Manuele Margni

With/Avec: Monsieur F. Bergeron; Dr. A. Desjardins; Professeur J. Guertin; Monsieur É. Hufty; Monsieur P. Lachapelle; Prof. N. Lahrichi; Madame M. Mallet; Mr. S. Mikhaylin

Institution paid/ Établissement payé: Université Laval

Title/Titre: Étude de l'éco-efficience du port de l'équipement de protection individuelle contre la COVID-19 dans les urgences pour un contexte post-pandémique

Primary Inst./Inst. principal: Santé publique et des populations
Other Related Inst./ Autres inst. connexes: Services et politiques de la santé

Competition /Concours: Subvention Projet
April/Avril 01, 2021

Number in competition/Nbre de demandes dans le concours: 2381

Peer Review Committee Recommendation, for your information and use/
Recommandation du comité d'examen par les pairs, pour fins d'information et d'utilisation:

**Committee/Comité:**	Santé publique, santé communautaire et santé des populations 3
**Number reviewed/ Demandes examinées:**	39
**Application rank within the committee/ Rang de la demande dans le comité:**	3
**Percent Rank within the committee / Rang en pourcentage au sein du comité:**	94.74%
**Rated / Cote:**	4.43
**Recommended Term/ Durée recommandée:**	2 years/ans 6 months/mois
**Recommended average annual operating amount/ Montant annuel moyen recommandé pour le fonctionnement:**	$84,000
**Recommended equipment amount/ Montant recommandé pour les appareils:**	$0

This document is for information only.
An application rated below 3.50 is ineligible for CIHR funding. For applications rated 3.50 and above, please note that it is the application's rank within the peer review committee that determines whether it is funded, rather than its absolute rating. The final funding decision will be communicated in the Notice of Decision.

Document à titre d'information seulement.
Une demande cotée en dessous de 3,5 n'est pas admissible au financement des IRSC. En ce qui a trait aux demandes cotées 3,50 ou plus, veuillez noter que l'on détermine l'attribution des fonds en fonction du classement obtenu au sein du comité d'examen par les pairs plutôt qu'en fonction du classement absolu. La décision finale relative au financement sera communiquée dans l'Avis de décision.

**Review Type / Type d'évaluation:**	Reviewer 1 / Évaluateur 1
**Name of Applicant / Nom du chercheur:**	Berthelot, Simon
**Application No. / Numéro de demande:**	462760
**Agency / Agence:**	CIHR/IRSC
**Competition / Concours:**	Project Grant/Subvention Projet
**Committee / Comité:**	Public, Community & Population Health 3/Santé publique, santé communautaire et santé des populations 3
**Title / Titre:**	Étude de l'éco-efficience du port de l'équipement de protection individuelle contre la COVID-19 dans les urgences pour un contexte post-pandémique

Adjudication Criteria/Critères de sélection

Initial Score/Cote Initiale: 4.2

Top/Bottom Selection/Groupe supérieur/inférieur

- (●) **Top/Groupe supérieur**
- ( ) **Bottom/Groupe inférieur**

**Review Type / Type d'évaluation:**	Reviewer 1 / Évaluateur 1
**Name of Applicant / Nom du chercheur:**	Berthelot, Simon
**Application No. / Numéro de demande:**	462760
**Agency / Agence:**	CIHR/IRSC
**Competition / Concours:**	Project Grant/Subvention Projet
**Committee / Comité:**	Public, Community & Population Health 3/Santé publique, santé communautaire et santé des populations 3
**Title / Titre:**	Étude de l'éco-efficience du port de l'équipement de protection individuelle contre la COVID-19 dans les urgences pour un contexte post-pandémique

Summary of Application/Résumé de la demande:

L’hypothèses générale est qu’avec la vaccination de masse et l’amélioration de plusieurs composantes de prévention des infections incluses dans la hiérarchie des mesures, les risques de transmission de la COVID19 seront réduits significativement et que le port de l’ÉPI selon les règles actuelles aura des conséquences économiques et écologiques dépassant les bénéfices anticipés.

Les objectifs de recherche principaux sont d’estimer:

1) Les bénéfices cliniques (réduction de la transmission, des admissions, de la mortalité et de l’absentéisme) pour les professionnel.le.s de la santé (immuns ou non immuns) de l’utilisation de l’ÉPI contre la COVID-19 ;

2) Le coût financier de l’utilisation de l’ÉPI à l’urgence pour les soins des personnes suspectées d’avoir la COVID-19;

3) L’empreinte écologique de l’utilisation de l’ÉPI à l’urgence pour les soins des personnes suspectées d’avoir la COVID-19.

**Review Type / Type d'évaluation:**	Reviewer 1 / Évaluateur 1
**Name of Applicant / Nom du chercheur:**	Berthelot, Simon
**Application No. / Numéro de demande:**	462760
**Agency / Agence:**	CIHR/IRSC
**Competition / Concours:**	Project Grant/Subvention Projet
**Committee / Comité:**	Public, Community & Population Health 3/Santé publique, santé communautaire et santé des populations 3
**Title / Titre:**	Étude de l'éco-efficience du port de l'équipement de protection individuelle contre la COVID-19 dans les urgences pour un contexte post-pandémique

Strengths and Weaknesses/Forces et faiblesses:

Approche de recherche

Forces:

Le candidat fait un compte-rendu très clair des incertitudes et recommandations parfois conflictuelles sur l'utilisation de l'EPI dans les hôpitaux et ce faisant dresse un portrait du besoin de données probantes à ce sujet, pour informer (et convaincre) les professionnels de la sante ainsi que permettre aux gestionnaires d'établissements de sante de prendre des décisions informées par des données scientifiques complètes.

Pour la revue systématique, le port de l'EPI standard sera considéré séparément du port de l'EPI renforce. Le travail sur cette revue systématique a déjà été amorce.

Les phases 1, 2 et 3 sont bien décrites et semblent prendre en ligne de compte les facteurs importants.

Faiblesses:

Pour l'évaluation du temps dédié a chaque action, il semble que plusieurs estimes sont sujet à être variables, dépendamment de l'expérience du personnel, l'heure du jour, l'achalandage de l'urgence, etc. Même un estime erroné de quelques secondes ou minutes pourrait largement influencer les calculs de cout s'il s'applique à une action très fréquente.

D'autres couts sociaux ou intangibles de l'usage d'EPI dans les urgences tels que les erreurs/manques de communication quand le visage et ses expressions ne peuvent être interprètes, la réduction du contact humain entre le patient et les intervenants, l'inconfort et le stress induits par le port de l'EPI, etc. Ne sont pas vraiment considères.

Pour la phase 4, les groupes de discussion, le nombre de 6 participants par groupe semble bas pour permettre une représentation adéquate (saturation des données) des différents niveaux (purposeful sampling strategy) étant donne le nombre important de niveaux devant être considérés. Il semble également que les discussions au sein de ces groupes seront susceptibles d'être dominées par les personnalités plus fortes, ce qui pourrait facilement masquer certaines perspectives si représentées par une seule personne au tempérament plus efface. De plus ample évidence justifiant 6 individus par groupe airait été désirable. L'approche proposée de recruter des groupes supplémentaires au besoin est discutable.

Comment les participants des groupes de discussion prendront-ils connaissance des résultats de l'étude? Sont-ils présentés avec un synopsis par les chercheurs de l'étude? Liront-ils un rapport écrit? Cette prise de connaissance se fera-t-elle avant ou durant la rencontre visioconférence? Comment les participants sont-ils identifiés et invites? De plus amples détails seraient nécessaires. Le but de cet exercice n'est pas clair et risque d'être indument influencé par la façon de présenter les résultats.

Originalité de la proposition

Forces:

Les clinicien.ne.s et les gestionnaires disposent de peu d'outils pour comprendre et agir sur l'empreinte écologique de leurs services et ce projet de recherche génèrera des données pertinentes sur l'utilisation de l'EPI.

L'approche multidisciplinaire 360 du port de l'EPI est ambitieuse et originale. En effet, l'étude de l'empreinte écologique est une approche intéressante qui est rarement considérée au sein de la recherche biomédicale mais qui semble à la fois logique et avant-gardiste.

Les méthodes proposées semblent appropriées. La phase 1 (revue systématique) est moins originale mais nécessaire. La

phase 2 (étude des couts) est basée sur une approche compréhensive qui tient compte des couts réels, incluant les couts caches ou moins évidents, comme le temps dédié aux activités reliées à l'EPI tel l'habillage/déshabillage, désinfection, le nettoyage des EPI réutilisables, etc.

Faiblesses:

Candidats

Forces:

The Candidat est un chercheur en début de carrière en médecine familiale et médecine d'urgence à l'Université Laval. Il est Assistant Professeur à l'Université Laval depuis 2015. Il est Co-candidat sur plusieurs fonds CIHR et a reçu des fonds de recherche du CMA et FMRQ, ainsi que deux projets de $100,000 du CIHR en tant que Candidat Principal.

Il est productif, ayant publié 6 articles en 2021 (2 en tant que 1er auteur, 2 dernier auteur, surtout dans les journaux spécialises en médecine d'urgence), et a contribué à 13 articles en 2020. H index 10

Il a supervisé 3 étudiants maitrise et un post-doc (pas de PhD a date).

Il a deux Co-candidats principaux qui sont eux en milieu de carrière, 8 Co-candidats de plusieurs institutions au Québec, ainsi que plusieurs utilisateurs de connaissance listes en tant que collaborateurs.

Le candidat, bien que médecin d'urgence, a déjà une expertise acquise sur l'étude de l'empreinte écologique et le « cycle de vie » dans le domaine de la sante.

Faiblesses:

Le rôle des patients partenaires à l'intérieur du projet n'est pas clairement établi ou défini.

L'équipe est nombreuse et peu de détails sont fournis quant à la gestion du projet et de l'équipe. Y aurait-il des réunions? Si oui a quelle fréquence? Quel sera le rôle des étudiants? Les expertises de chaque appliquant/collaborateur est décrite mais leur rôle dans l'étude est parfois nébuleux. Il n'est pas clair que le candidat principal a le temps ou l'expertise de dirige rune équipe si nombreuse et il n'y a pas d'indication qu'il est encadre par d'autres candidats dans ce rôle.

Environment de la recherche

Forces:

Les cinq urgences du CHU Québec utilisent des EPI et protocoles similaires et reçoivent plus de 240,000 visites annuellement, en faisant un excellent environnement pour une étude telle que celle proposée.

Le projet a reçu le support de plusieurs organisations partenaires, avec lettres de support enthousiastes à l'appui. Le candidat a accès aux données nécessaires.

Faiblesses:

Peu d'étudiants sont impliqués dans le projet et leur rôle n'est pas clair. Quel sera le rôle d l'étudiant€ au PhD? Peu de formation intégrée au projet.

Il serait désirable de savoir si les coordinatrices et assistantes de recherche a temps partiel sont déjà à l'emploi et ont l'expérience de travailler au CHU ou si elles devront être engagées.

Impact de la recherché

Forces :

Le projet est séduisant et adresse un besoin important dans le domaine de la santé ou du système de soins de santé.

Les résultats sur l’éco-efficience devrait facilement être généralisables à l’ensemble de la province et même du pays.

Le potentiel est définitivement présent pour un impact important sur la future utilisation de l’EPI dans les urgences (et hôpitaux en général) du Québec et d’ailleurs.

Faiblesses:

L’emphase de l’impact des résultats semble être ciblée sur les soins d’urgence alors que plusieurs résultats pourraient être relevant à l’ensemble des soins de santé.

Des exemples décrivant “présentations pour le grand public dans des forums ou congrès citoyens” seraient utiles.

**Review Type / Type d'évaluation:**	Reviewer 1 / Évaluateur 1
**Name of Applicant / Nom du chercheur:**	Berthelot, Simon
**Application No. / Numéro de demande:**	462760
**Agency / Agence:**	CIHR/IRSC
**Competition / Concours:**	Project Grant/Subvention Projet
**Committee / Comité:**	Public, Community & Population Health 3/Santé publique, santé communautaire et santé des populations 3
**Title / Titre:**	Étude de l'éco-efficience du port de l'équipement de protection individuelle contre la COVID-19 dans les urgences pour un contexte post-pandémique

Budget Recommendation/Recommandation budgétaire:

Le budget semble bas.

Please indicate your appraisal of the integration of sex as a biological variable as a strength, weakness, or not applicable to the proposal./Prière de sélectionner une option pour donner votre évaluation de l'intégration du sexe comme variable biologique en tant que point fort ou point faible de la proposition, ou en tant qu'élément non applicable à la proposition.

- (●) **Strength/Point fort**
- ( ) **Weakness/Point faible**
- ( ) **Not applicable/Non applicable**

Please indicate your appraisal of the integration of gender as a socio-cultural determinant of health as a strength, weakness, or not applicable to the proposal./Prière de sélectionner une option pour donner votre évaluation de l'intégration du genre comme déterminant socioculturel de la santé en tant que point fort ou point faible de la proposition, ou en tant qu'élément non applicable à la proposition.

- ( ) **Strength/Point fort**
- (●) **Weakness/Point faible**
- ( ) **Not applicable/Non applicable**

**Review Type / Type d'évaluation:**	Reviewer 1 / Évaluateur 1
**Name of Applicant / Nom du chercheur:**	Berthelot, Simon
**Application No. / Numéro de demande:**	462760
**Agency / Agence:**	CIHR/IRSC
**Competition / Concours:**	Project Grant/Subvention Projet
**Committee / Comité:**	Public, Community & Population Health 3/Santé publique, santé communautaire et santé des populations 3
**Title / Titre:**	Étude de l'éco-efficience du port de l'équipement de protection individuelle contre la COVID-19 dans les urgences pour un contexte post-pandémique

Sex and/or Gender Considerations/Notions de sexe et/ou de genre:

Les candidats stipulent que le sexe et le genre seront considères dans chaque objectif. Cependant, aucun détail n’est donné quant à méthode utilisée pour déterminer le genre a l’intérieur de l’étude.

**Review Type / Type d'évaluation:**	Reviewer 2 / Évaluateur 2
**Name of Applicant / Nom du chercheur:**	Berthelot, Simon
**Application No. / Numéro de demande:**	462760
**Agency / Agence:**	CIHR/IRSC
**Competition / Concours:**	Project Grant/Subvention Projet
**Committee / Comité:**	Public, Community & Population Health 3/Santé publique, santé communautaire et santé des populations 3
**Title / Titre:**	Étude de l'éco-efficience du port de l'équipement de protection individuelle contre la COVID-19 dans les urgences pour un contexte post-pandémique

Adjudication Criteria/Critères de sélection

Initial Score/Cote Initiale: 4.6

Top/Bottom Selection/Groupe supérieur/inférieur

- (●) **Top/Groupe supérieur**
- ( ) **Bottom/Groupe inférieur**

**Review Type / Type d'évaluation:**	Reviewer 2 / Évaluateur 2
**Name of Applicant / Nom du chercheur:**	Berthelot, Simon
**Application No. / Numéro de demande:**	462760
**Agency / Agence:**	CIHR/IRSC
**Competition / Concours:**	Project Grant/Subvention Projet
**Committee / Comité:**	Public, Community & Population Health 3/Santé publique, santé communautaire et santé des populations 3
**Title / Titre:**	Étude de l'éco-efficience du port de l'équipement de protection individuelle contre la COVID-19 dans les urgences pour un contexte post-pandémique

Summary of Application/Résumé de la demande:

Le projet porte sur l’utilisation d’équipements de protection individuelle à l’urgence suite à la pandémie de COVID-19. On anticipe que malgré les taux de vaccination qui montent et le risque de contamination réduit, l’utilisation d’équipements de protection individuelle persiste. L’équipe souhaite donc évaluer la pertinence de maintenir cette utilisation dans les services d’urgences.

Le projet comporte quatre étapes permettant respectivement : 1) d’estimer l’effet de l’équipement de protection individuelle utilisée dans les services d’urgences hospitalières afin de protéger le personnel médicale contre les infections à la COVID-19, 2) d’estimer les coûts associés à l’utilisation d’équipements de protection individuelle, 3) d’estimer l’impact environnementale de l’utilisation d’équipements de protection individuelle, et 4) de mettre en relation les bénéfices, coûts et impacts d’équipements de protection individuelle et en discuter avec différents groupes d’intérêts pour orienter la prise de décision sur leurs utilisations.

La première étape repose sur une revue systématique, la deuxième étape, sur une évaluation économique qui considère à la fois le coût matériel, mais aussi les coûts indirects particulièrement liées à la gestion des équipements et le temps associé à leurs utilisations, la troisième étape repose sur une analyses des émissions et ressources associées aux produits impliqués dans l’approvisionnement, l’utilisation, et la disposition vers les rebuts ou la préparation à une réutilisation des équipements de protection individuelle. Finalement, la quatrième étape permet une analyse coût-conséquences en comparant les économies et bénéfices cliniques que les équipements de protection individuelle auront permis de produire (réduction d’hospitalisation, absentéisme, etc) aux coûts associés à leurs utilisations et leurs impacts environnementales.

Il s’agit d’une proposition fort novatrice menée par une équipe multidisciplinaire détenant toutes les expertises requises pour mener à bien le projet. En plus d’inclure des chercheurs avec des compétences et expériences complémentaires, l’équipe inclut plusieurs utilisateurs de connaissances et patients partenaires. Ceux-ci ont été inclus dans le projet dès le début et continueront d’être impliqué tout au long du processus, favorisant ainsi le développement d’objectifs d’importance pour les gestionnaires et directeurs de programmes, d’institutions et d’unités de soins. Cette approche représente aussi une excellente stratégie pour que les résultats soient réellement implantés en pratique. Ceci est un excellent exemple d’approche intégrée de transfert de connaissances alors qu’elle se positionne pour avoir un impact réel sur le processus décisionnel en matière d’approvisionnement et utilisation d’équipement de protection individuelle.

**Review Type / Type d'évaluation:**	Reviewer 2 / Évaluateur 2
**Name of Applicant / Nom du chercheur:**	Berthelot, Simon
**Application No. / Numéro de demande:**	462760
**Agency / Agence:**	CIHR/IRSC
**Competition / Concours:**	Project Grant/Subvention Projet
**Committee / Comité:**	Public, Community & Population Health 3/Santé publique, santé communautaire et santé des populations 3
**Title / Titre:**	Étude de l'éco-efficience du port de l'équipement de protection individuelle contre la COVID-19 dans les urgences pour un contexte post-pandémique

Strengths and Weaknesses/Forces et faiblesses:

Le projet sera appliqué aux cinq services d'urgence du centre hospitalier universitaire de l'Université Laval à Québec. Même si ce centre n'est peut-être pas représentatif de tout le Canada ou le Québec, il s'agit du plus grand centre hospitalier au Québec et d'un des trois plus grand au Canada. De plus, on peut présumer que les protocoles utilisés à ce CHU sont similaires à ce qu'on retrouve ailleurs et que les fournisseurs sont les mêmes à d'autres CHU ou du moins ont un impact environnemental similaire.

La revue de littérature en phase 1 part d'une méthodologie qui a déjà été utilisée dans le contexte des épidémies de SARS et de MERS. Toutes les étapes sont menées par deux évaluateurs indépendants pour éviter les erreurs. Il y a un risque que la revue de littérature ne relève pas d'études sur le sujet. Ceci est toutefois peu probable vu l'intérêt de la communauté scientifique sur tout ce qui a trait à la COVID-19. Néanmoins, s'il manque de littérature pour atteindre l'objectif d'estimer l'effet de l'équipement de protection individuelle utilisée dans les services d'urgences hospitalières afin de protéger le personnel médical contre les infections à la COVID-19, l'équipe prévoit faire des extrapolations de ses résultats pour l'appliquer à sa population d'intérêt. Peu de détails sont présentés sur cette stratégie d'atténuation. Il est donc difficile de juger de son adéquation, pourtant essentielle pour assurer l'objectif ultime de l'étude de permettre aux décideurs de prendre une décision basée sur une description juste et détaillée des coûts et conséquences. NOTE qu'on comprends quand même que le risque de ne pas trouver suffisamment de littérature est minime.

Il est mentionné que les discussions à l'étape 4 (visant à faire une analyse coût-conséquences en comparant les économies et bénéfices cliniques que les équipements de protection individuelle auront permis de produire aux coûts associés à leurs utilisations et leurs impacts environnementales) seront basées sur des résultats présentés séparément de façon non agrégée. Ceci aura l'avantage de permettre aux participants de déterminer pour eux-mêmes le poids et la valeur qu'ils attribueront aux coûts et aux conséquences présentés en vue d'une prise de décision ou de position éclairée. Il serait toutefois pertinent de discuter de seuils sur lesquels d'autres auraient pris des décisions dans le passé.

L'équipe multidisciplinaire de ce projet, la distribution de rôles parmi des chercheurs de niveaux d'expérience variés, le positionnement de membres de l'équipe de recherche dans des groupes d'influence, l'intégration sincère d'utilisateurs de connaissances et patients partenaires, le plan de transfert de connaissances, le potentiel d'influencer la pratique et l'adhésion aux principes de recherche axée sur le patient sont des forces importantes de ce projet.

**Review Type / Type d'évaluation:**	Reviewer 2 / Évaluateur 2
**Name of Applicant / Nom du chercheur:**	Berthelot, Simon
**Application No. / Numéro de demande:**	462760
**Agency / Agence:**	CIHR/IRSC
**Competition / Concours:**	Project Grant/Subvention Projet
**Committee / Comité:**	Public, Community & Population Health 3/Santé publique, santé communautaire et santé des populations 3
**Title / Titre:**	Étude de l'éco-efficience du port de l'équipement de protection individuelle contre la COVID-19 dans les urgences pour un contexte post-pandémique

Budget Recommendation/Recommandation budgétaire:

Le budget proposé est raisonnable et bien arrimé avec les objectifs et le plan de travail.

Please indicate your appraisal of the integration of sex as a biological variable as a strength, weakness, or not applicable to the proposal./Prière de sélectionner une option pour donner votre évaluation de l'intégration du sexe comme variable biologique en tant que point fort ou point faible de la proposition, ou en tant qu'élément non applicable à la proposition.

- (●) **Strength/Point fort**
- ( ) **Weakness/Point faible**
- ( ) **Not applicable/Non applicable**

Please indicate your appraisal of the integration of gender as a socio-cultural determinant of health as a strength, weakness, or not applicable to the proposal./Prière de sélectionner une option pour donner votre évaluation de l'intégration du genre comme déterminant socioculturel de la santé en tant que point fort ou point faible de la proposition, ou en tant qu'élément non applicable à la proposition.

- (●) **Strength/Point fort**
- ( ) **Weakness/Point faible**
- ( ) **Not applicable/Non applicable**

**Review Type / Type d'évaluation:**	Reviewer 2 / Évaluateur 2
**Name of Applicant / Nom du chercheur:**	Berthelot, Simon
**Application No. / Numéro de demande:**	462760
**Agency / Agence:**	CIHR/IRSC
**Competition / Concours:**	Project Grant/Subvention Projet
**Committee / Comité:**	Public, Community & Population Health 3/Santé publique, santé communautaire et santé des populations 3
**Title / Titre:**	Étude de l'éco-efficience du port de l'équipement de protection individuelle contre la COVID-19 dans les urgences pour un contexte post-pandémique

Sex and/or Gender Considerations/Notions de sexe et/ou de genre:

L’intégration de la notion de sexe et de genre est adéquate.

**Review Type / Type d'évaluation:**	Reviewer 3 / Évaluateur 3
**Name of Applicant / Nom du chercheur:**	Berthelot, Simon
**Application No. / Numéro de demande:**	462760
**Agency / Agence:**	CIHR/IRSC
**Competition / Concours:**	Project Grant/Subvention Projet
**Committee / Comité:**	Public, Community & Population Health 3/Santé publique, santé communautaire et santé des populations 3
**Title / Titre:**	Étude de l'éco-efficience du port de l'équipement de protection individuelle contre la COVID-19 dans les urgences pour un contexte post-pandémique

Adjudication Criteria/Critères de sélection

Initial Score/Cote Initiale: 4.4

Top/Bottom Selection/Groupe supérieur/inférieur

- (●) **Top/Groupe supérieur**
- ( ) **Bottom/Groupe inférieur**

**Review Type / Type d'évaluation:**	Reviewer 3 / Évaluateur 3
**Name of Applicant / Nom du chercheur:**	Berthelot, Simon
**Application No. / Numéro de demande:**	462760
**Agency / Agence:**	CIHR/IRSC
**Competition / Concours:**	Project Grant/Subvention Projet
**Committee / Comité:**	Public, Community & Population Health 3/Santé publique, santé communautaire et santé des populations 3
**Title / Titre:**	Étude de l'éco-efficience du port de l'équipement de protection individuelle contre la COVID-19 dans les urgences pour un contexte post-pandémique

Summary of Application/Résumé de la demande:

Le projet proposé vise à mesurer les bénéfices cliniques pour les prestataires de soins, les coûts et l'empreinte écologiques de l'utilisation des équipements de protection individuelle (EPI) dans les salles d'urgence du CHUQ, dans un contexte où la population sera largement vaccinée contre la COVID-19. Le projet inclue 4 phases : 1) revue de littérature systématique des bénéfices cliniques de l'EPI; 2) une estimation des coûts de l'utilisation de l'EPI; 3) l'estimation de de l'empreinte écologique de l'EPI durant la pandémie; et 4) analyses coûts conséquences et de groupes de discussion.

**Review Type / Type d'évaluation:**	Reviewer 3 / Évaluateur 3
**Name of Applicant / Nom du chercheur:**	Berthelot, Simon
**Application No. / Numéro de demande:**	462760
**Agency / Agence:**	CIHR/IRSC
**Competition / Concours:**	Project Grant/Subvention Projet
**Committee / Comité:**	Public, Community & Population Health 3/Santé publique, santé communautaire et santé des populations 3
**Title / Titre:**	Étude de l'éco-efficience du port de l'équipement de protection individuelle contre la COVID-19 dans les urgences pour un contexte post-pandémique

Strengths and Weaknesses/Forces et faiblesses:

Forces

•La considération de l'impact écologique des interventions médicales est innovateur, actuel et pertinent. L'EPI est essentielle pour bien protéger les travailleurs de la santé, mais son usage doit demeurer justifié, et l'impact de son utilisation au niveau des coûts et de l'environnement vont pouvoir mieux guider son usage dans le futur. L'EPI affecte aussi grandement l'expérience des patients à l'urgence ou dans les hôpitaux, alors une optimisation de leur utilisation (sélective mais sécuritaire) pourrait aussi bénéficier aux patients.

•Il sera très bien de connaître quel a été l'impact de l'utilisation de l'EPI pendant la pandémie, mais les connaissances acquises par ce projet permettront aussi de mieux guider l'usage de l'EPI dans le futur. Les ajustements des indications de EPI ont varié grandement pendant la pandémie, il semble nécessaire de solidifier les connaissances dans ce domaine pour usage futur. L'impact potentiel de ce projet est donc considéré important.

•L'équipe est excellente, et a clairement l'expertise pour mener ce projet avec succès. Le chercheur principal est nouveau chercheur, mais pratique à l'urgence depuis 20 ans.

•La faisabilité du projet présenté semble tout à fait réaliste. L'équipe est composée de plusieurs personnes avec expertises variées et pertinentes, incluant des patients partenaires.

•L'étude bénéficie d'un momentum actuel, mais devrait être réalisée relativement rapidement pour maintenir sa pertinence.

Faiblesses

•Malgré les forces décrites ci-haut, il demeure qu'il est possible que les données analysées dans la revue systématiques ne s'appliquent qu'au contexte particulier de la pandémie. Il y a donc un fort risque que les études varient grandement sur les équipements utilisés, la prévalence locale de la COVID-19 lors de l'étude, la pénurie d'équipement en cours à ce moment, etc, et que les conclusions soient difficiles à tirer ou peu applicable à un contexte de population largement vaccinée.

•Au-delà du bénéfice pour le travailleur de la santé, il serait intéressant d'examiner les bénéfices des différents EPI pour prévenir les éclosions dans les milieux de soins. Il est possible que certains EPI protègent contre les éclosions, ou encore mènent à un taux d'auto-contamination élevé lors du retrait ou du nettoyage, ou qu'une mauvaise utilisation mène à la contamination de l'environnement, ce qui aurait le potentiel de causer des éclosions dans les milieux de soins. Ce point est davantage une suggestion qu'une faiblesse.

**Review Type / Type d'évaluation:**	Reviewer 3 / Évaluateur 3
**Name of Applicant / Nom du chercheur:**	Berthelot, Simon
**Application No. / Numéro de demande:**	462760
**Agency / Agence:**	CIHR/IRSC
**Competition / Concours:**	Project Grant/Subvention Projet
**Committee / Comité:**	Public, Community & Population Health 3/Santé publique, santé communautaire et santé des populations 3
**Title / Titre:**	Étude de l'éco-efficience du port de l'équipement de protection individuelle contre la COVID-19 dans les urgences pour un contexte post-pandémique

Budget Recommendation/Recommandation budgétaire:

No concerns.

Please indicate your appraisal of the integration of sex as a biological variable as a strength, weakness, or not applicable to the proposal./Prière de sélectionner une option pour donner votre évaluation de l'intégration du sexe comme variable biologique en tant que point fort ou point faible de la proposition, ou en tant qu'élément non applicable à la proposition.

- (●) **Strength/Point fort**
- ( ) **Weakness/Point faible**
- ( ) **Not applicable/Non applicable**

Please indicate your appraisal of the integration of gender as a socio-cultural determinant of health as a strength, weakness, or not applicable to the proposal./Prière de sélectionner une option pour donner votre évaluation de l'intégration du genre comme déterminant socioculturel de la santé en tant que point fort ou point faible de la proposition, ou en tant qu'élément non applicable à la proposition.

- (●) **Strength/Point fort**
- ( ) **Weakness/Point faible**
- ( ) **Not applicable/Non applicable**

**Review Type / Type d'évaluation:**	Reviewer 3 / Évaluateur 3
**Name of Applicant / Nom du chercheur:**	Berthelot, Simon
**Application No. / Numéro de demande:**	462760
**Agency / Agence:**	CIHR/IRSC
**Competition / Concours:**	Project Grant/Subvention Projet
**Committee / Comité:**	Public, Community & Population Health 3/Santé publique, santé communautaire et santé des populations 3
**Title / Titre:**	Étude de l'éco-efficience du port de l'équipement de protection individuelle contre la COVID-19 dans les urgences pour un contexte post-pandémique

Sex and/or Gender Considerations/Notions de sexe et/ou de genre:

Adequate, but justification of considerations could be more specific as to the expected roles of sex and of gender in these analyses.

Review Type/Type d'évaluation: SO Notes /Notes de l'agent scientifique
Name of Applicant/Nom du chercheur: Berthelot, Simon
Application No./Numéro de demande: 462760
Agency/Agence: CIHR/IRSC
Competition/Concours: 2021-04-01 Project Grant/Subvention Projet
Committee/Comité: Public, Community & Population Health 3/Santé publique, santé communautaire et santé des populations 3
Title/Titre: Étude de l'éco-efficience du port de l'équipement de protection individuelle contre la COVID-19 dans les urgences pour un contexte post-pandémique

---

Assessment/Évaluation:

Competition: 202104PJT
PRC: PH3
NPA: Berthelot, Simon
Application Number: 462760
Project Title: Étude de l'éco-efficience du port de l'équipement de protection individuelle contre la COVID-19 dans les urgences pour un contexte post-pandémique

SO Notes begin here (please do not include this sentence or the text above in the final version of SO notes when uploading to ResearchNet):
````````````````````````````````````````````````````````````````````````````````````````````````````````````

## Strengths (including SGBA considerations):

The research question was considered an important topic to explore. The lack of data to guide PPE guidelines is an important problem to address and the applicants propose a practical and refreshing approach to addressing it. The project has potential for very significant impact.

This is a strong multi-disciplinary team with complementary skills and experience doing the specific components of the study as described.

The holistic and comprehensive approach to exploring and calculating financial and ecological costs is a strength.

The strong and well-articulated integrated KT including a wide range of stakeholders and including key knowledge users in every step of the project is a strength.

Including five large ERs in the project is a strength and makes the findings likely to be generalizable to others across the country.

The budget was very reasonable budget for the design.
````````````````````````````````````````````````````````````````````````````````````````````````````````````

Review Type/Type d'évaluation: SO Notes /Notes de l'agent scientifique
Name of Applicant/Nom du chercheur: Berthelot, Simon
Application No./Numéro de demande: 462760
Agency/Agence: CIHR/IRSC
Competition/Concours: 2021-04-01 Project Grant/Subvention Projet
Committee/Comité: Public, Community & Population Health 3/Santé publique, santé communautaire et santé des populations 3
Title/Titre: Étude de l'éco-efficience du port de l'équipement de protection individuelle contre la COVID-19 dans les urgences pour un contexte post-pandémique

---

Assessment/Évaluation:

Weaknesses (including SGBA considerations):

There was some concern that 24 participants may not be enough to reach saturation in the qualitative phase given the diversity of the participants in this phase of the study.

The previous literature that the applicants propose to analyze will likely be very inconsistent with respect to the context of PPE use; that will mean that the data to be analyzed is quite "messy" which may make it difficult to draw conclusions.

It would be interesting to address the costs of the difficulty of communicating while wearing PPE (for example this could be integrated into the qualitative phase).

More detail with respect to how they are considering sex and/or gender in the project would have been helpful.

Budget:

No changes noted.

****

Note: The final rating of the application, provided in the Notice of Recommendation (NOR) and Notice of Decision (NOD), is the averaged rating of the peer review committee members following the discussion of the application during the committee meeting, and therefore may differ from the ratings provided by the assigned reviewers in their respective reviews.

Remarque : La cote définitive de la demande, qui apparaît dans l'avis de recommandation et l'avis de décision, représente la moyenne des cotes accordées par les membres du comité d'évaluation par les pairs après avoir débattu de la demande à la réunion du comité. Elle peut donc différer de celle donnée par les évaluateurs dans leur évaluation respective.

**Review Type/Type d'évaluation:**	SO Notes /Notes de l'agent scientifique
**Name of Applicant/Nom du chercheur:**	Berthelot, Simon
**Application No./Numéro de demande:**	462760
**Agency/Agence:**	CIHR/IRSC
**Competition/Concours:**	2021-04-01 Project Grant/Subvention Projet
**Committee/Comité:**	Public, Community & Population Health 3/Santé publique, santé communautaire et santé des populations 3
**Title/Titre:**	Étude de l'éco-efficience du port de l'équipement de protection individuelle contre la COVID-19 dans les urgences pour un contexte post-pandémique

Assessment/Évaluation:

````````````````````````````````````````````````````````````````````````````````````````````````````````````````````````````````````````````````

***SO Notes end here (please do not include this sentence in final version of SO notes when uploading to ResearchNet).***
````````````````````````````````````````````````````````````````````````````````````````````````````````````````````````````````````````````````
